# Evolution of ColE1-like plasmids across γ-Proteobacteria: From bacteriocin production to antimicrobial resistance

**DOI:** 10.1371/journal.pgen.1009919

**Published:** 2021-11-30

**Authors:** Manuel Ares-Arroyo, Eduardo P. C. Rocha, Bruno Gonzalez-Zorn

**Affiliations:** 1 Antimicrobial Resistance Unit (ARU), Faculty of Veterinary Medicine and VISAVET, Complutense University of Madrid, Madrid, Spain; 2 Institut Pasteur, Université de Paris, CNRS, UMR3525, Microbial Evolutionary Genomics, Paris, France; Universidad de Sevilla, SPAIN

## Abstract

Antimicrobial resistance is one of the major threats to Public Health worldwide. Understanding the transfer and maintenance of antimicrobial resistance genes mediated by mobile genetic elements is thus urgent. In this work, we focus on the ColE1-like plasmid family, whose distinctive replication and multicopy nature has given rise to key discoveries and tools in molecular biology. Despite being massively used, the hosts, functions, and evolutionary history of these plasmids remain poorly known. Here, we built specific Hidden Markov Model (HMM) profiles to search ColE1 replicons within genomes. We identified 1,035 ColE1 plasmids in five Orders of γ-Proteobacteria, several of which are described here for the first time. The phylogenetic analysis of these replicons and their characteristic MOB_P5/HEN_ relaxases suggest that ColE1 plasmids have diverged apart, with little transfer across orders, but frequent transfer across families. Additionally, ColE1 plasmids show a functional shift over the last decades, losing their characteristic bacteriocin production while gaining several antimicrobial resistance genes, mainly enzymatic determinants and including several extended-spectrum betalactamases and carbapenemases. Furthermore, ColE1 plasmids facilitate the intragenomic mobilization of these determinants, as various replicons were identified co-integrated with large non-ColE1 plasmids, mostly via transposases. These results illustrate how families of plasmids evolve and adapt their gene repertoires to bacterial adaptive requirements.

## Author summary

The extraordinary adaptability of bacteria and the massive prevalence of mobile genetic elements within populations has turned antimicrobial resistance into a growing threat to Public Health. Among all the mobile genetic elements, plasmids have been the focus of attention as these extrachromosomal molecules of DNA are able to mobilize several antimicrobial resistance genes at once through conjugation. However, although small mobilizable and non-conjugative replicons have been traditionally overlooked when analyzing plasmid-mediated antimicrobial resistance, they have recently been described as important carriers of AMR genes. In this work, we have analyzed the ColE1-like plasmid family, whose study has been neglected even if they are one of the main groups of small plasmids in natural populations of Proteobacteria. We observed that these plasmids have evolved for a long time within γ-Proteobacteria acquiring different genetic features in specific hosts, being major players in the spread of antimicrobial resistance determinants.

## Introduction

Plasmids are extrachromosomal self-replicating molecules of DNA able to transfer between bacteria mainly by conjugation [1]. They play a crucial role in bacterial evolution as they are key drivers of horizontal gene transfer, the major process of gene repertoire variation in prokaryotes [2]. Moreover, plasmids usually encode antimicrobial resistance determinants among their cargo genes and are considered to be the main spreaders of resistance in clinical environments [3].

Among their extraordinary diversity, there is a family of plasmids that has become very popular due to its widespread use in biotechnology since the 1970s: the ColE1-like plasmids (*ColE1 plasmids* hereinafter) [4]. Their history is closely related to the history of colicin-like bacteriocins, as pColE1 got its name by being the first plasmid characterized encoding the colicin E1 [5]. Since then, the ColE1-like group of replicons refers to every plasmid whose mechanism of replication resembles the original plasmid pColE1, most of which have been related to colicin production. All these plasmids share the same characteristics, traditionally described as small, multicopy and mobilizable replicons [6], generally associated to the MOB_P5/HEN_ family of relaxases [7]. Recently, we showed that these small multicopy plasmids are encapsidated in phages with up to 10,000 times more efficiency than large plasmids, suggesting that phages could be major vectors of antimicrobial resistance genes borne in ColE1 plasmids [8].

The extended popularity of ColE1 plasmids in biotechnology lies in their ability to be stably maintained at high copy number within the cell due to their characteristic mechanism of replication mediated by two antisense and overlapping RNAs encoded in the origin of replication or *ori* [9]. Briefly, the ~550 bp RNA II pre-primer binds to its homologous DNA forming an RNA-DNA hybrid that triggers plasmid replication [10]. This mechanism is regulated by the ~100 bp RNA I, transcript that forms three stem loops complementary to the nascent structure of RNA II, to which it binds forming the *kissing complex* (RNA I-RNA II). This union modifies the secondary structure of the RNA II, inhibiting its binding to the plasmid DNA, thus, impeding the plasmid replication [11]. Some ColE1 plasmids encode an auxiliary protein called Rop (*Repressor of primer*) or Rom (*RNA One Modulator*), which stabilizes the *kissing complex* [12].

Known ColE1 replicons show a narrow host-range, mostly restricted to the Order *Enterobacterales*, where they were first described and extensively analyzed in terms of molecular biology [5,9]. In contrast, their role in wild-type populations has remained poorly studied [13], with a few works suggesting their presence in other γ-Proteobacteria [14–20]. Notwithstanding, recent studies have shown that small multicopy plasmids can have an enormous impact on bacterial evolution [21], often related to the dissemination and evolution of antimicrobial resistance determinants [13,15,22–25].

Given the increasing urgence in understanding the vectors of antimicrobial resistance, we have identified and studied the diversity of this overlooked family of plasmids. We combined a ColE1 Hidden Markov Model (HMM) profile of our own with PlasmidFinder [26] to identify ColE1 plasmids within the RefSeq database. We successfully collected 1,035 replicons and explored, for the first time, the evolutionary history of the ColE1 family among different Orders of γ-Proteobacteria focusing on both the ColE1 origin of replication and its MOB_P5/HEN_ relaxase. This revealed the co-evolution of different mechanisms of replication within some ColE1 plasmids and its association with plasmid size. Finally, we examined the functional contribution that ColE1 replicons provide to their host, highlighting their role in the dissemination of antimicrobial resistance.

## Results and discussion

### ColE1 replicons are spread across five Orders of Proteobacteria

**Fig 1:**
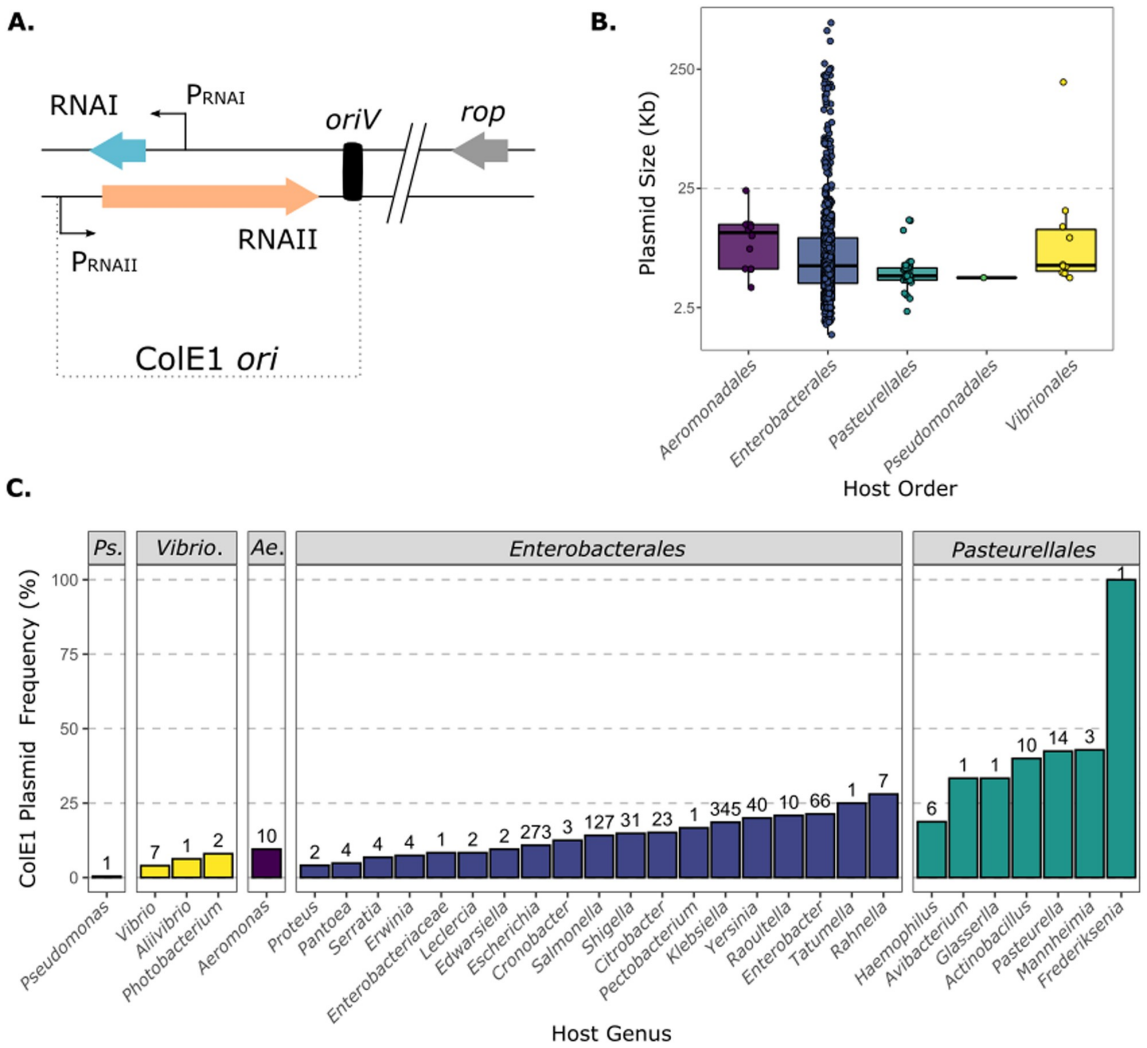
Identification of ColE1 plasmids. **A. The ColE1 origin of replication**. Schematic representation of the ColE1 *ori*, including the RNAI, RNAII, their promoters and the *oriV* site. The gene encoding the auxiliary protein Rop is also represented. **B. Size of ColE1 plasmids**. Size of the ColE1 replicons attending to their Order. The y-axis represents the size(Kb) in logarithmic scale. **C. Frequency of ColE1 plasmids within the Genus plasmidome**. Proportion of ColE1 among plasmids of each Genus of bacteria. The x-axis represents the Genus, whereas the y-axis the frequency of ColE1 plasmids among all the plasmids from the Genus (%). The numbers at the top of the bars indicate the absolute number of ColE1 plasmids identified. The figure includes only the replicons identified in RefSeq. *Ps*.: Pseudomonadales. *Vibrio*.: Vibrionales. *Ae*.: Aeromonadales.

To identify ColE1 plasmids, we constructed two HMM profiles based on the sequence of 81 ColE1 replicons described in the literature (S1 Table). One profile includes the whole ~550 bp ColE1 origin of replication (*ori*), from the RNA II promoter to the origin of replication site (*oriV*), whereas the second one includes only the ~100 bp RNA I (Fig 1A). As the origin of replication of ColE1 plasmids from *Pasteurellales* was still uncharacterized, we studied eight ColE1 replicons from this Order to build specific HMM profiles (S1 Text).

Using the aforementioned HMM profiles and PlasmidFinder (see Materials and methods), we searched the 20,532 plasmids available in RefSeq and identified 1,003 ColE1 plasmids. PlasmidFinder proved to be highly efficient in the identification of these replicons, as 884 out of the 1,003 plasmids were correctly identified as ColE1. Still, our HMM profiles successfully identified 126 additional ColE1 plasmids, substantially increasing the sensitivity of the search. Indeed, they were crucial for broadening the host spectrum of ColE1 replicons, as 96.5% of plasmids outside *Enterobacterales* were exclusively identified with the HMM profiles. Additionally, 32 ColE1 plasmids used for the construction of the profiles were not present in the RefSeq database. The final dataset has 1,035 ColE1 plasmids, with a mean average size of 14.7 kb and a median of 5.6 kb (Fig 1B and S2 Table).

The replicons were found in 33 different genera, 11 families and 5 Orders of γ-Proteobacteria. Most plasmids were identified in *Enterobacterales*, with *Klebsiella* and *Escherichia* accounting for 60.5% of all presently identified ColE1 plasmids. This is largely due to the over-representation of these bacteria in the database (Fig 1C). ColE1 plasmids represent 18.6% and 10.8% of all known plasmids from *Klebsiella* spp. and *Escherichia* spp., whereas they account for almost half of the plasmids in major representatives of *Pasteurellales*. In *Aeromonadales* and *Vibrionales* ColE1 are 9.5% and 6.1% of all plasmids, respectively. Interestingly, two ColE1 plasmids were identified in *Pseudomonadales*, one in *Pseudomonas* and another in *Acinetobacter*. At this stage it is thus unclear if these plasmids are rare in *Pseudomonadales* or if our method lacks sensitivity to identify them. We conclude that ColE1 plasmids are very abundant across at least four Orders of γ-Proteobacteria, showing particularly high prevalence within *Pasteurellales*.

**Fig 2:**
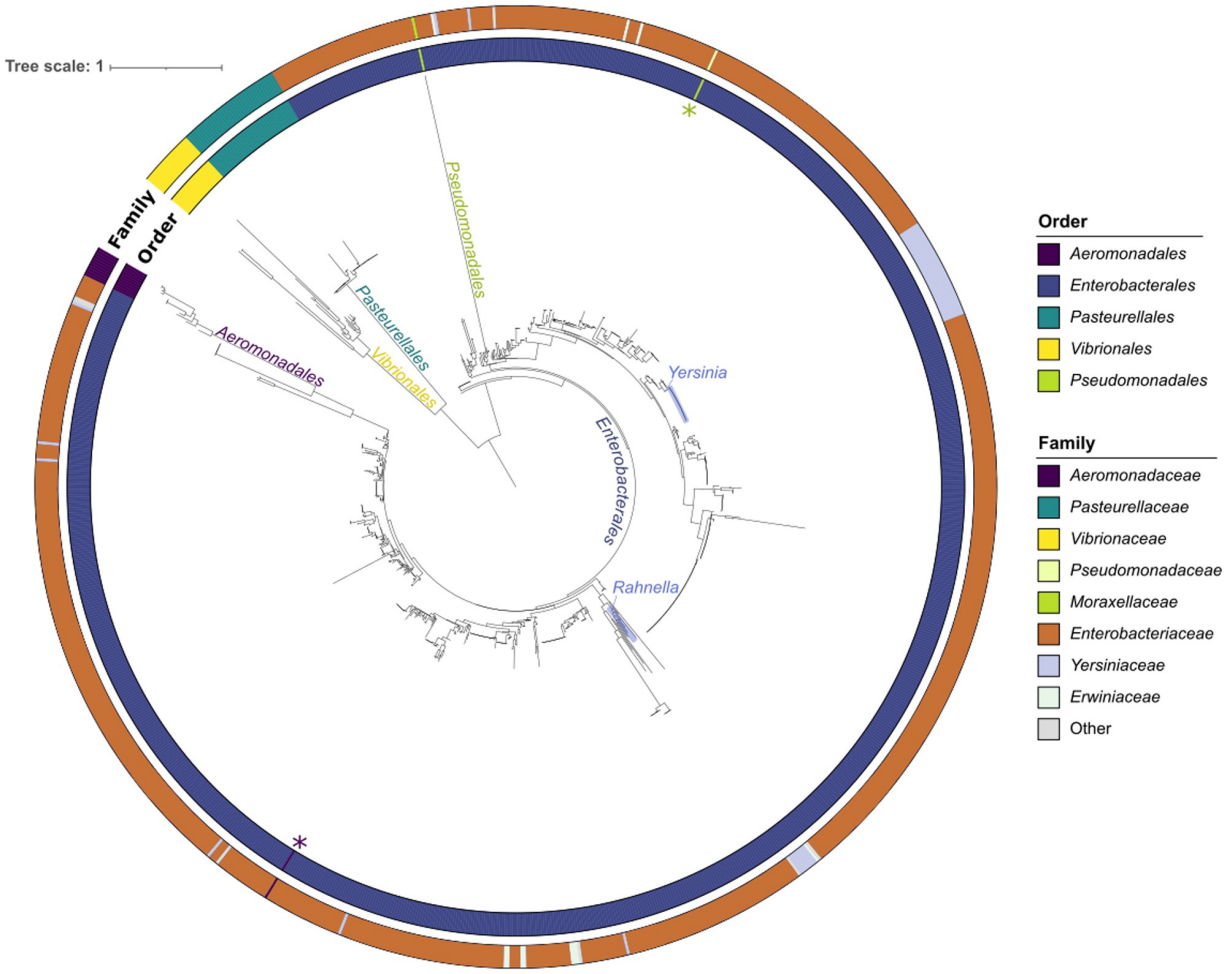
Phylogenetic tree of the 1,035 ColE1 origins of replication. The colors of the inner circle represent the Order in which the replicon was identified, whereas the outer circle indicates the Family. The two asterisks next to the inner circle shows the two plasmids clustered within the clade of another Order. The phylogenetic tree was inferred following the best-fit model, SYM+R7.

Some distinctions in the ColE1 *ori* among host clades have already been described. While the RNAs involved in replication generate three stem loops in *Enterobacterales* [27] and *Aeromonadales* [20], they generate only two in *Vibrionales* [16] and *Pasteurellales* (S1 Text). To assess the evolutionary relations between the ColE1 replicons, we constructed a phylogenetic tree of the 1,035 ColE1 origins of replication, defined as the region encoded from the RNA II promotor to the *oriV* site. Despite nucleotide sequences being worse phylogenetic markers than proteins [28], the tree was robust enough to observe a clear separation between replicons of different Orders. Even if it clusters the *Aeromonadales* and *Pseudomonadales* within the *Enterobacterales* clade, these correspond to very long branches whose basal position is not very well supported (Fig 2). Only two plasmids out of the 1,035 (0.19%) were classed within other Orders. In contrast, plasmids from different genera were often close together in the phylogenetic tree (S1–S4 Figs), suggesting frequent transfer between bacteria of different genera. Hence, plasmids seem unable to transfer across Orders, but often transfer across genera.

### Co-evolution of the ColE1 *ori* and putative Rep proteins

**Fig 3:**
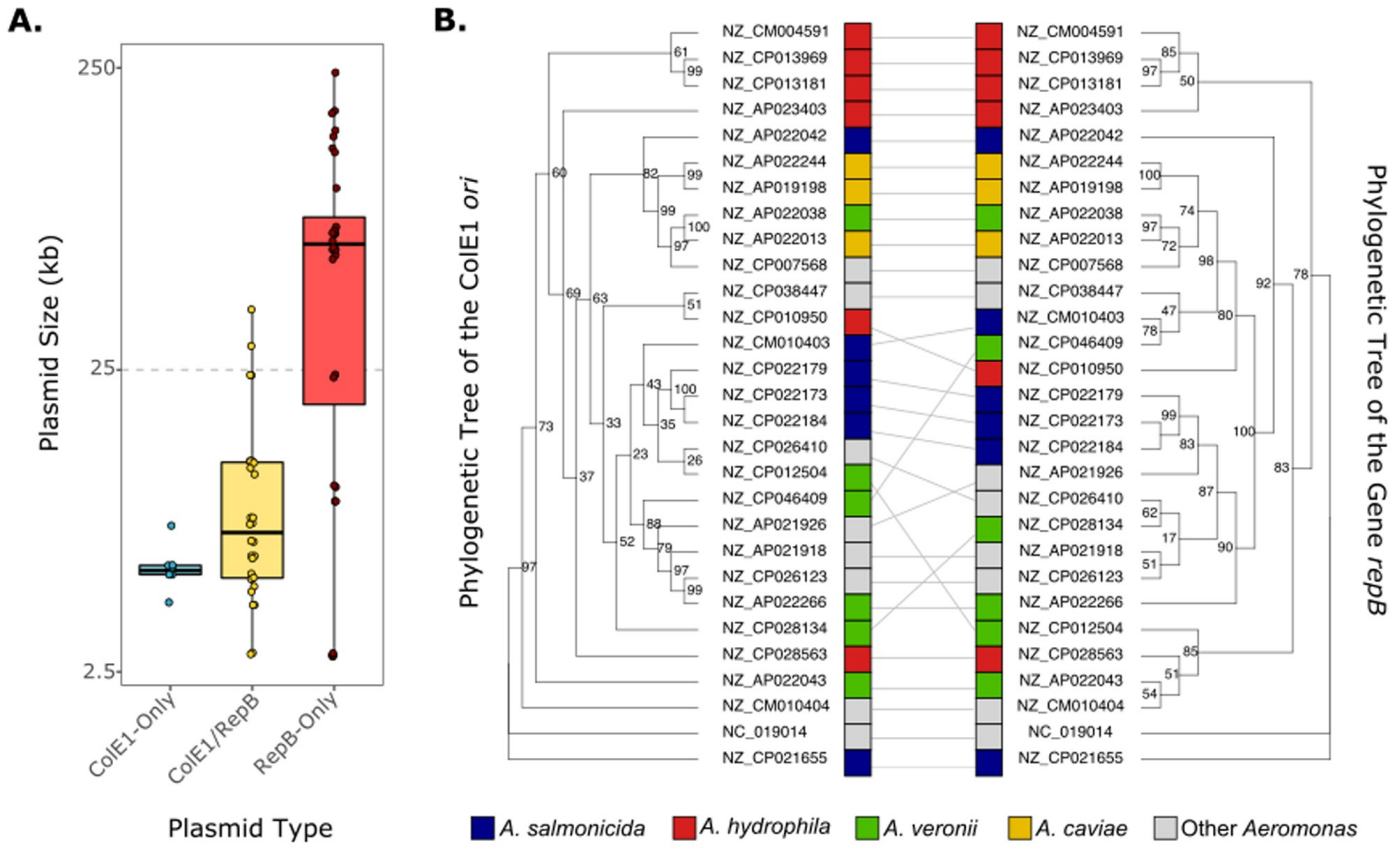
ColE1/RepB plasmids in *Aeromonadales*. **A**. Size of the ColE1/RepB plasmids (log scale) per category: ColE1-only (n = 8), ColE1/RepB (n = 30) and RepB-only (n = 28). The horizontal gray dashed line separates the small (<25kb) from the large plasmids (>25kb). **B**. Tanglegram of the ColE1 origin of replication (left) (best-fit model JTT+G4) and the gene *repB* (right) (TN+F+I+G4). The bootstrap values of the trees are indicated with numbers next to each node. The accession numbers and host Species of the plasmids are indicated at the tips of the branches.

As the ColE1 mechanism of replication does not require any Rep protein, the presence of small ColE1/Rep plasmids in *Pasteurellales* and *Aeromonadales* was intriguing (S2 Table). In *Pasteurellales*, the ColE1 *ori* association with *rep* genes seems to have occurred through different independent events (S2 Text). In *Aeromonadales*, we identified two different plasmids from distinct sources and hosts (*A*. *hydrophila* and *A*. *salmonicida*) encoding a similar ColE1 *ori* and a putative RepB protein (81.5% and 82.0%, pairwise identity, respectively). Although this RepB protein is annotated as *RepB replication protein* in databases (RefSeq reference: WP_103859311.1) and it has been associated to plasmid replication in the literature [29,30], we have not found experimental evidence of its function. We will refer to it as putative RepB. To verify if this combination is a common phenomenon in this Order, and due to their small representation in our 1,035 ColE1 collection, we collected additional plasmids of *Aeromonadales* from RefSeq Assemblies, filtering those sequences encoding a ColE1 *ori* and/or the RepB protein. We obtained 4 chromosomes and 68 plasmids (S3 Table). Among plasmids, 8 encode just the ColE1 origin of replication (*ColE1-only* hereinafter), 32 the *repB* gene (*RepB-only*), and 28 both (*ColE1/RepB*) (Fig 3A). Hence, ColE1 plasmids in *Aeromonadales* are more frequently found with *repB* than alone.

The 8 ColE1-only plasmids are small (*μ* = 5,555 bp) (Fig 3A), whereas the RepB-only plasmids tend to be much larger (*μ* = 71,947 bp). Interestingly, plasmids with both elements are small (*μ* = 10,448 bp), and only slightly larger than the ColE1-only (t = 2.897, df = 29, p-value = 0.007). This finding denotes that *repB* is common among small ColE1 and large non-ColE1 plasmids within *Aeromonadales*. However, the phylogenetic tree of all the ColE1 *ori* from *Aeromonadales* separate the ColE1-only from the ColE1/RepB (S5 Fig), suggesting a unique *repB* acquisition/loss event. Indeed, their combination seems to be important for the plasmid as both elements show a strong genetic linkage, *repB* being usually in the immediate kilobase upstream the ColE1 *ori* (S6 Fig). To confirm this hypothesis, we built a tanglegram of the phylogenetic trees of the two genes ColE1/RepB. This analysis revealed their remarkable similarity, highlighting conserved clades of plasmids from *A*. *hydrophila*, *A*. *caviae/A*. *veronii*, *A*. *salmonicida* and other low-represented species (Figs 3B and S7). Hence, our results suggest that both elements have been co-evolving in plasmids moving between diverse species of *Aeromonadales*. Therefore, ColE1 plasmids show an alternative evolutionary trajectory within this Order, frequently encoding a putative replication gene but conserving the ColE1 origin of replication itself.

### Genesis and evolution of ColE1 co-integrates

Although plasmids containing diverse types of replicons are common [31–33] and co-integration between small and large plasmids is known to occur [34–37], there is limited information available on the genesis and evolution of ColE1 co-integrates with large plasmids. Among the 1,035 ColE1 collection, 64 plasmids were larger than 25 kb (*μ* = 118.7 kb) (Fig 1B), which suggests a co-integration of the ColE1 plasmid with larger ones. We used PlasmidFinder to identify additional non-ColE1 plasmid types in the 62 “circular” ones. We found them in 33 of the 62 plasmids, mostly from the IncC, IncFIA, IncFIB, IncFII, IncN, IncN2 and IncN3 groups (S2 Table). We evaluated if these plasmids were co-integrates by looking for ColE1 related genes and the ColE1 *ori* in these larger plasmids. In many cases we identified the auxiliary gene *rop*, bacteriocin production operons or antimicrobial resistance determinants and transposons typically identified in ColE1-like plasmids (S9 Fig). Therefore, our analysis revealed that the co-integration of ColE1 with other plasmids is frequent. Of note, although many of these plasmids were previously described, their ColE1 origin of replication remained unnoticed [38–43].

**Fig 4:**
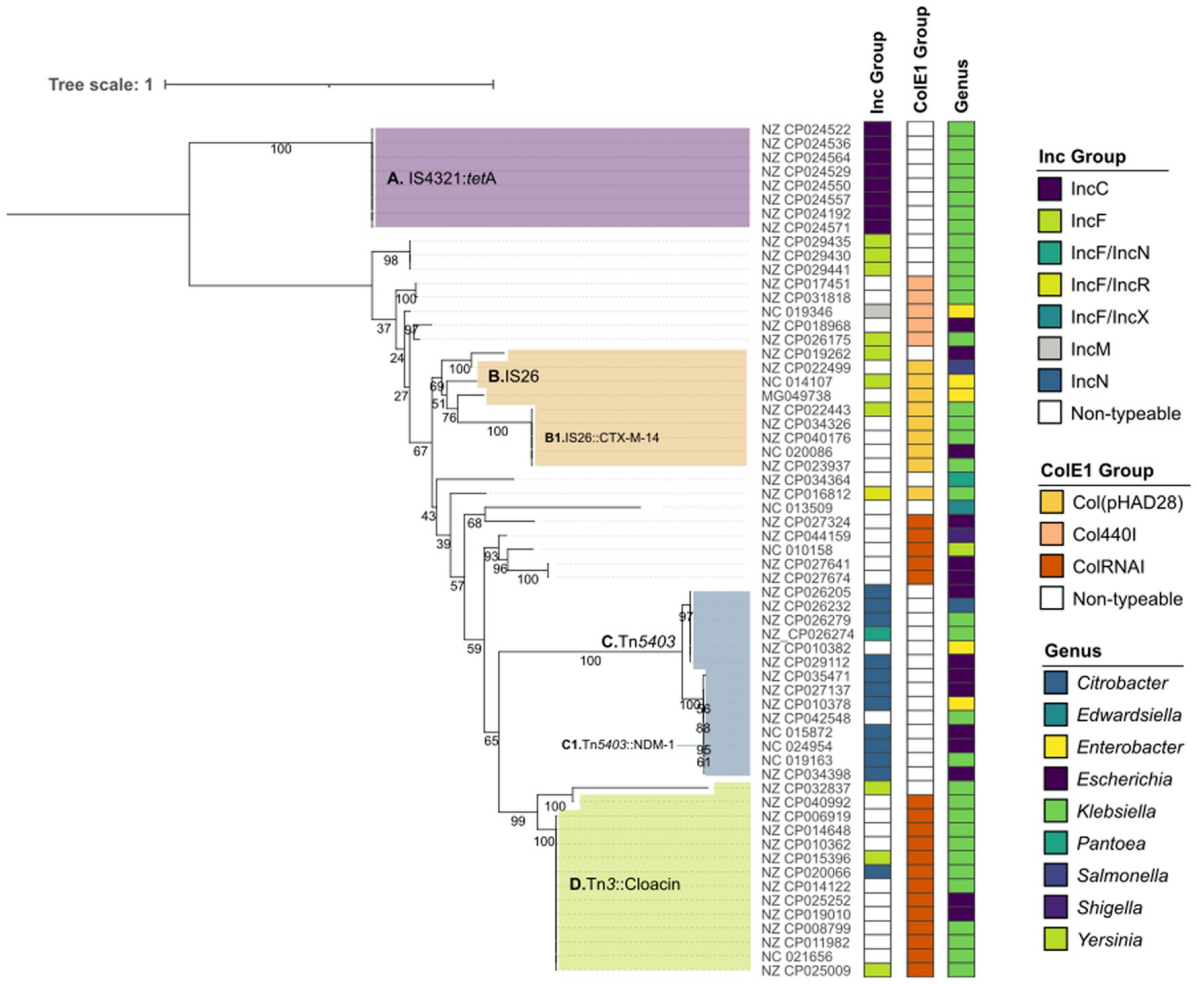
Phylogenetic tree of the ColE1 origins of replication in large plasmids (>25kb) from *Enterobacterales*. The bootstrap values are indicated with a number next to each node. The accession number of the 62 plasmids are indicated at the right of the tree. The color of the columns next to the accession numbers shows, from the left to the right, the Inc group associated to the large plasmid (identified with PlasmidFinder), the ColE1 origin of replication (identified with PlasmidFinder) and the host Genus. The colored shades within the tree (A-D) represent different patterns of co-integration exhibited by the plasmids, further detailed in the S9 Fig. Next to the letter A-D, it is indicated the putative transposon responsible for the recombination event. The phylogenetic tree was inferred following the best-fit model, K2P+I+G4.

Among the 62 putative ColE1 co-integrates, 61 belonged to the Order *Enterobacterales*. The exception was a 194,647 bp plasmid from *Vibrio campbellii* (NZ_CP026317.1), non-typeable by PlasmidFinder. The co-integration in this plasmid occurred immediately upstream an *rpn*-like endonuclease (S8 Fig), which is the candidate responsible for the recombination event [44]. In *Enterobacterales*, the tree of the 61 ColE1 origins of replication tends to cluster co-integrates according to the existence of additional replicon types (*e*.*g*. the IncC and IncN clades), albeit there are exceptions (*e*.*g*. the IncF replicons) (Fig 4). To evaluate if this distribution was the result of unrelated recombination events or a co-evolution process of the ColE1 *ori* and the additional replicon, we analyzed the most represented clusters of co-integrates (S9 Fig): clade A (ColE1/IncC), clade B (ColE1/IncF and ColE1/NT), clade C (ColE1/IncN) and clade D (mostly ColE1/NT). Each of these clades represent co-integrates generated by different recombinases (Fig 4 and S3 Text).

The results show varied patterns of genesis and evolution of the co-integrates. In some cases, the conserved genetic environment surrounding the ColE1 *ori* suggests a co-integration event and subsequent co-evolution of the plasmids over time. These are the cases of the ColE1/IncC (clade A) and ColE1/IncN (clade C). The origin of the former clade seems to be recent, having been produced by a single recombination event involving IS4321s. The latter represents a successful association, as the co-integration through a Tn*5403* has been conserved and spread among different hosts (*e*.*g*. *Escherichia*, *Klebsiella*, *Enterobacter*, *Citrobacter*) (Fig 4). In contrast, the genetic environment of the ColE1 *ori* in other clades suggests that the integration resulted from independent recombination events. That is the case of cluster D, in which the co-integration has occurred in diverse single events with various plasmid types (IncF, IncN, NT) through a Tn*3* transposase and mobilizing a whole colicin operon. Lastly, cluster C shows an intermediate situation, in which different recombination events though an IS26 have occurred in different plasmids and hosts, but generating a successful co-integrate that has been evolving in *Escherichia* and *Klebsiella* (Cluster B1, Fig 4). Interestingly, most successful associations involve the mobilization of antimicrobial resistance genes (clade A: *tetA*; clade B1: *bla*_CTX-M-14_; clade C: *bla*_NDM-1_) and will be further discussed below.

### The MOB_P5/HEN_ relaxase has co-evolved with the ColE1 *ori* among different Orders while influenced by *rop*

To better understand the evolution of ColE1 plasmids in relation to conjugation, we analyzed its MOB_P5/HEN_ relaxase [7,45]. As it has been noticed that many plasmids lack the relaxase [15], we first investigated its prevalence after discarding the 62 putative co-integrates and the 82 incomplete sequences. Ca. 39% of the ColE1 plasmids encode a relaxase with large differences across Orders: ~90% in *Pasteurellales*, ~40% in *Enterobacterales* and *Aeromonadales*, none in *Vibrionales* (S2 Table). Among the 348 ColE1 plasmids carrying a relaxase, we identified 352 relaxases of which most were MOB_P_ (n = 339) bearing the characteristic motif III of the MOB_P5/HEN_ group. Two plasmids carried different relaxases (MOB_Q_ and MOB_V_) and 11 had truncated relaxase genes.

**Fig 5:**
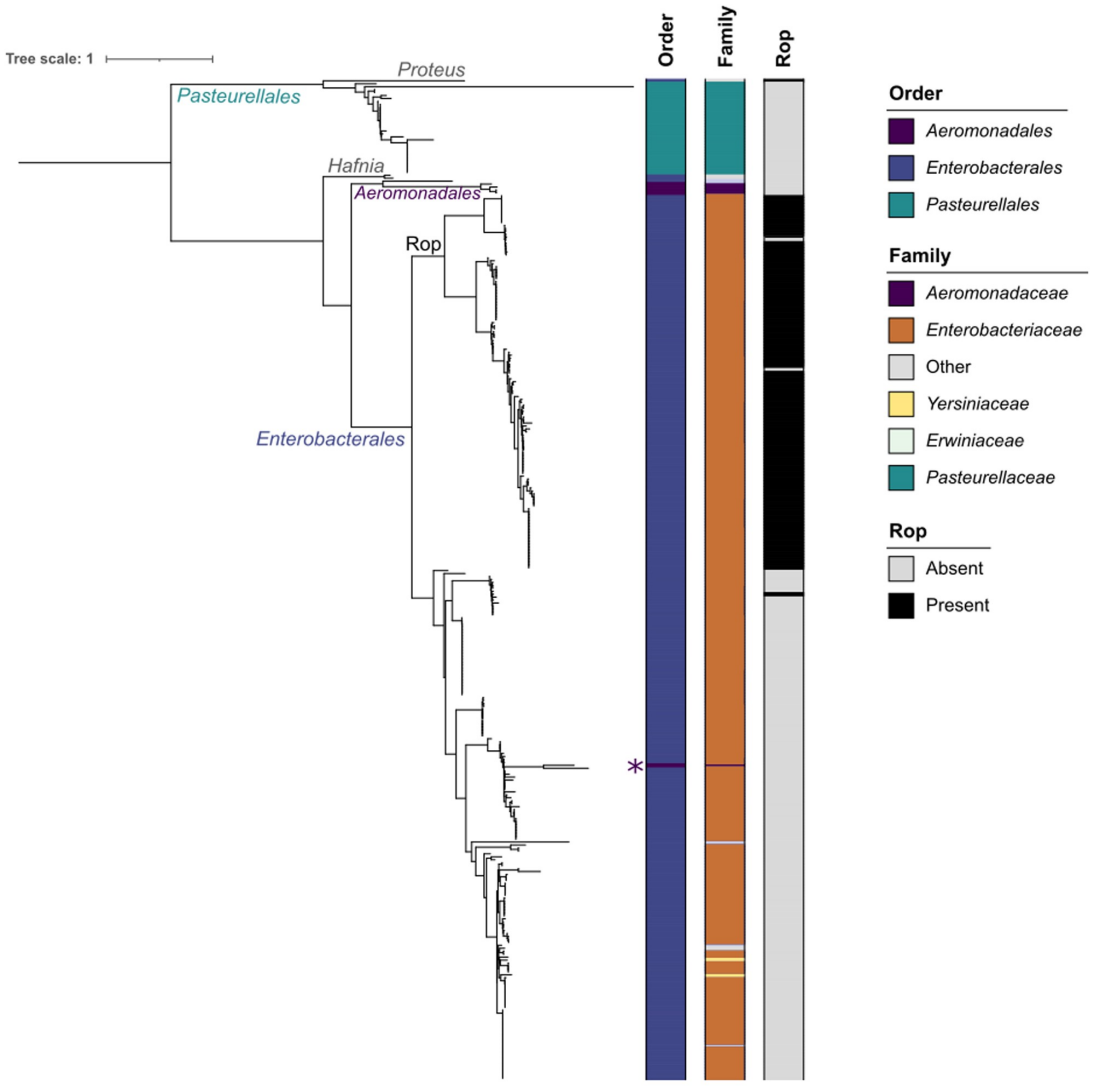
Phylogenetic tree of the 339 MOB_P5/HEN_ relaxases. The colors of the columns at the right of the tree indicate, from the left to the right, the host Order, the host Family and the presence or absence of the *rop* gene in the plasmid. The asterisk next to the first column at the right of the tree represents the only relaxase clustered with plasmids of other Order. The phylogenetic tree was inferred following the best-fit model, JTT+F+I+G4.

We built a phylogenetic tree of the MOB_P5/HEN_ relaxases encoded in the plasmids (Fig 5). The tree clusters the proteins by the host Order even clearer than the ColE1 *ori* (Fig 2), implying a different evolutionary trajectory within each Order. Within Orders, the relaxase does not cluster at the genus-level (S10–S12 Figs). The large *Enterobacteriaceae* clade is divided into two groups, one of them constituted mostly by *Escherichia* plasmids, whereas the other clade included a diverse group of bacteria. The distinctive characteristic between the two clades is the presence of the auxiliary replication gene *rop* in the plasmid (Fig 1A), which is encoded in 59% of the plasmids from *Enterobacterales* (Figs 5 and S12). Although *rop* is negatively associated with the presence of a relaxase (X^2^ = 82.057, df = 1, p < 2.2e-16), their combination is not rare in the genus *Escherichia*, mainly associated to ColRNAI and Col(pHAD28) replicons (S12 Fig). This finding is consistent with previous works that have postulated plasmid recombination events through the *oriT* and *cer* sites [46,47], which are located at the opposite ends of the *rop*-relaxase genetic region [15]. This result implies that the relaxase and *rop* might be co-evolving within specific genera of *Enterobacterales* moving between different plasmids and mediating the evolution of this family of replicons.

### The functional contribution of ColE1 plasmids

The ColE1 collection, after discarding plasmids with a “linear” status and putative co-integrates (n = 889), has 3,618 protein genes (*μ* = 4.07 genes/plasmid) and about 0.62 genes/kb (S4 Table). This is a lower gene density than usually found in bacterial chromosomes, >0.85 genes/kb [48], which may result from the existence of RNA genes or larger regulatory regions in the plasmids. Indeed, we could not identify a known protein coding gene in 50 ColE1 plasmids.

**Fig 6:**
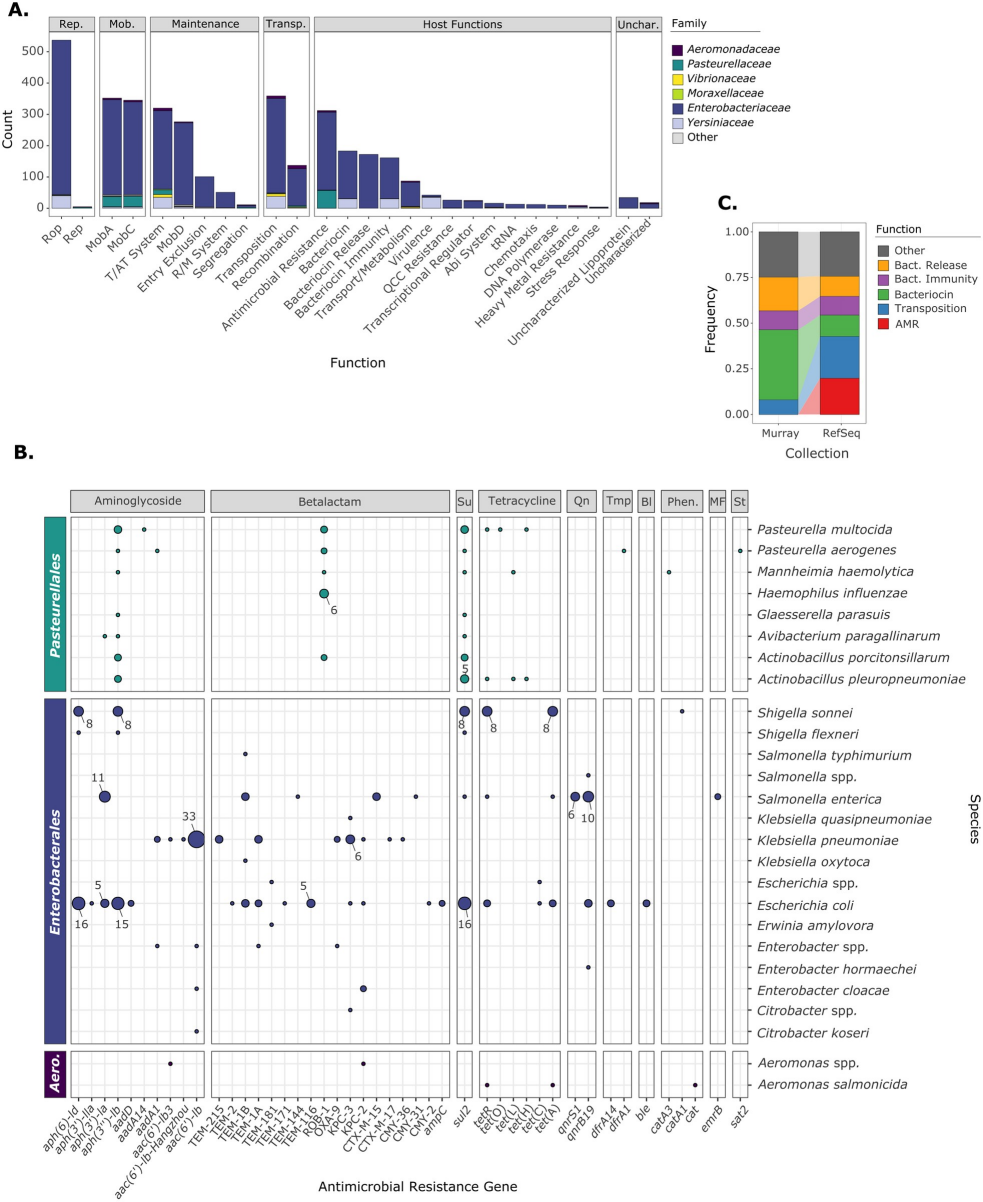
Functional classification of ColE1 plasmids. **A**. Functions encoded on the ColE1 plasmids. Only plasmids with a status “Complete” and “Circular” were retrieved. Putative co-integrates were discarded. Rep.: Replication. Mob.: Mobilization. Transp.: Transposition. Unchar.: Uncharacterized. T/AT System: Toxin/Antitoxin System. R/M System: Restriction/Modification System. QCC Resistance: Quaternary Cation Compound Resistance. Abi System: Abortive-Infection System. **B**. Antimicrobial resistance determinants in ColE1 plasmids. The circles within the figure indicate the presence of the gene, their size being proportional to the number of genes. When a gene was identified 5 or more times, the exact number is indicated next to the circle. *Aero*.: Aeromonadales. Su.: Sulphonamide. Qn.: Quinolone. Tmp.: Trimethoprim. Bl.: Bleomycin. MF.: MFS transporter. St.: Streptothricin. **C**. Shift in the cargo genes of ColE1 plasmids from the Pre-antibiotic Era (Murray Collection) to the current one (RefSeq collection). Only the functions classified as “Host Functions” and “Transposition” are represented.

The ColE1 genetic repertoire has 261 different gene families (S4 Table), which we classified into 6 categories and 27 subcategories (Fig 6A). Functions associated with plasmid biology, replication and mobilization, are the most represented(Rop, MobA, MobC, MobD). Toxin/antitoxin systems and transposases are also very frequent, notably the Tn*3* family (S4 Table). Nevertheless, ColE1 plasmids also present a variety of genes providing potential advantages to their host, some related to cell metabolism, virulence, defense from phages or heavy metal resistance. It was not unexpected to identify the production of colicin-like bacteriocins as one of the major functions provided by ColE1 plasmids [49], with 173 colicin-encoding plasmids from 9 different genera, despite these genes being restricted to plasmids of *Enterobacterales*. In contrast, it was surprising to find that antimicrobial resistance is the most frequent accessory function present in ColE1 plasmids (Fig 6A).

### Antimicrobial resistance encoded in ColE1 plasmids

The analysis of the ColE1-associated resistome showed that 20% (n = 182) of ColE1 plasmids harbor at least one antimicrobial resistance determinant for a total of 312 genes related to antimicrobial resistance (*μ* = 1.71 genes/plasmid). Eleven plasmids encode five or more antimicrobial resistance genes, frequently providing multidrug-resistance genotypes despite their small size. Interestingly, resistance determinants are preferentially encoded in ColE1 plasmids without relaxases (χ^2^ = 6.305, df = 1, p = 0.012), where they show a higher density (*μ* = 1.88 genes/plasmid) than in the relaxase-encoding plasmids (*μ* = 1.34 genes/plasmid).

The ColE1-associated resistome is represented by 45 different genes conferring resistance against 9 classes of antimicrobials (Fig 6B), mostly aminoglycosides (n = 127) and betalactams (n = 73). The majority of these genes encode for enzymatic determinants, such as betalactamases, aminoglycoside phosphotransferases or aminoglycoside acetyltransferases. Genes coding for enzymatic determinants show a dose-dependent phenotype and could benefit from the high copy numbers of ColE1 plasmids as they will amplify their expression [50]. Even more, betalactamases and aminoglycoside enzymatic determinants exhibit a wide range of variants [51,52] and multicopy plasmids have been demonstrated to potentiate the evolution of their plasmid-encoded genes as they provide with higher supply of mutations [23,53]. Therefore, it raises the question of whether they could be involved in the wide range of variants within these families of resistance genes.

Our analysis also revealed that ColE1 replicons are associated with the emergence of antimicrobial resistant species categorized by the WHO as “high priority pathogens for the research and development of new antibiotics” [54]: ampicillin-resistant *H*. *influenzae (bla*_ROB-1_), fluoroquinolone-resistant *Salmonella* (*qnrS* and *qnrB1*), and carbapenem-resistant and ESBL-producing *Enterobacteriaceae* (Fig 6B). Among the ESBLs and carbapenemases, we identified KPC-2, KPC-3, CTX-M-5, CTX-M-17, OXA-9, CMY-2, CMY-31 and CMY-36 encoded on ColE1 plasmids (Fig 6B). This study corroborates the growing evidence connecting the small multicopy replicons with ESBL and carbapenemase production observed in diverse isolates over the last years [13,55–59].

Although ColE1 co-integrates have not been included in the functional analysis above, their role in the evolution of antimicrobial resistance is worth mentioning. The co-integration of a CTX-M-17-encoding ColE1 plasmid with a large replicon has been already identified in a clinical *E*. *coli* from Vietnam and, furthermore, a recent study has observed that the 80% of KPC-3 producing *K*. *pneumoniae* outside hospital environments in Portugal carried the betalactamase in ColE1/IncF co-integrates [36,60]. Our results reveal that the co-integration of ColE1 plasmids is a widespread phenomenon in *Enterobacterales*, in many cases mediated by ESBL/carbapenemase-encoding transposons (NDM-1, CTX-M-14, KPC-2, KPC-3) (S9 Fig and S3 Text). Among these, the ColE1/IncN2 encoding NDM-1 and the ColE1/NT encoding CTX-M-14 seem to be the most relevant from a clinical perspective, as they have been identified in isolates from diverse Genera of *Enterobacterales* and spread across different continents [38,41,42]. These results demonstrate that ColE1 plasmids are key players in the mobility of antimicrobial resistance determinants within and between bacteria.

As ColE1 plasmids have been traditionally identified encoding bacteriocins, we wondered if AMR genes were recently acquired by this plasmid family. To do so, we compared our ColE1 collection against 115 ColE1 replicons identified within the Murray Collection (S5 Table, Materials and methods), *Enterobacteriaceae* isolates from the Pre-Antibiotic Era [61]. Their phylogenetic analysis cluster the ColE1 replicons from the Murray Collection together within our ColE1 collection (S13 Fig), showing little differences in terms of the ColE1 *ori*. However, none of the ColE1 plasmids from the Murray Collection encoded antimicrobial resistance (S6 Table), hence, the acquisition of AMR genes in ColE1 plasmids supposes a major recent shift in their cargo genes (Fig 6C) most certainly due to the increased selection pressure for acquisition of antibiotic resistance during the last decades.

## Conclusions and perspectives

**Fig 7:**
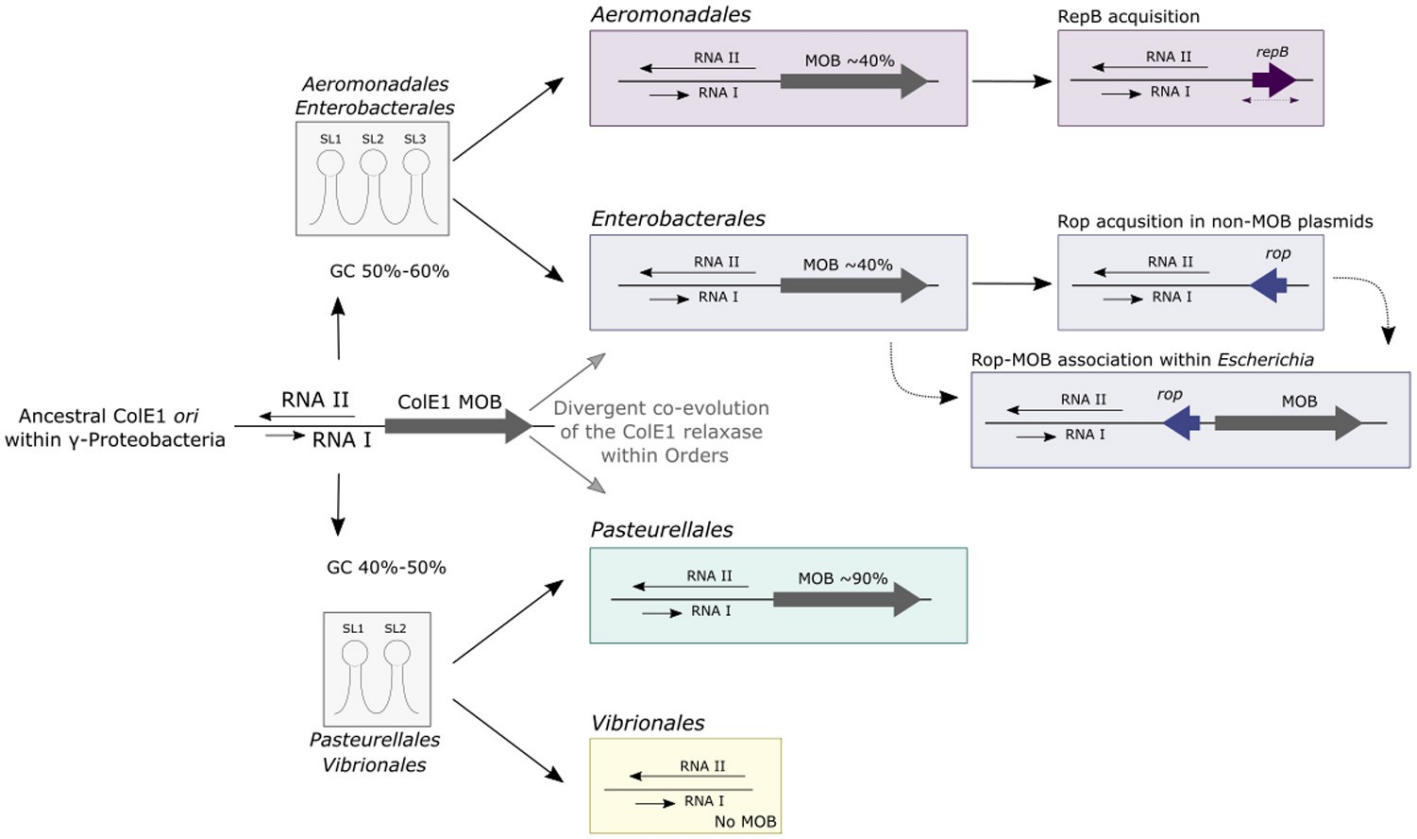
Evolutionary history of the ColE1 plasmids. The schematic representation of the ColE1 *ori* is illustrated with two antisense black arrows for both the RNAI and RNAII, and the ColE1 relaxase with a thick grey arrow annotated as MOB. The divergent evolution of the ColE1 *ori* within *Aeromonadales*/*Enterobacterales* and *Pasteurellales*/*Vibrionales*, respectively, is represented with black arrows ending in two grey boxes, each of them showing three (SL1-3) or two stem loops (SL1-2). At the right of the figure, the genetic elements related to the ColE1 plasmids within each Order are represented. The *rop* gene in *Enterobacterales* is represented with a blue arrow; the *repB* gene in *Aeromonadales* with a purple arrow; and the dashed purple arrow below the *repB* gene represents its variable location within the plasmid.

The present work provides new insights into the origin, evolution and current role of the ColE1-like plasmid family. The phylogeny of the ColE1 *ori* (Fig 2) and its MOB_P5/HEN_ relaxase (Fig 5) denotes key differences according to the Order in which they have been described. Their GC contents differ between clades because they resemble those of their hosts. For instance, the average GC in *Pasteurellales* is 41.5% for the ColE1 *ori*, 43.1% for the relaxase and 40.3% for their genome, whereas in *Enterobacterales* it is 52.8%, 57.7% and 53.0%, respectively (S2 Table). This is consistent with the phylogenetic evidence and indicates that the ColE1 origin of replication originated some time ago in the Class γ-Proteobacteria, where it has been divergently co-evolving with the MOB_P5/HEN_ relaxase within Orders but with little transfer across them (Fig 7). During this process, the secondary structure of the *kissing complex* has been modified and additional genes have been acquired in some taxa, such as *repB* in *Aeromonadales* or *rop* in *Enterobacterales*, the latter further associated with the relaxase in *Escherichia* (Fig 7). Nevertheless, our phylogenetic analysis suggests that although the ColE1 *ori* are specific to each Order, plasmids transfer much more freely across Genera (S1–S4 and S10–S12 Figs).

Additionally, we have observed a functional shift of cargo genes between ColE1 plasmids identified prior to the extended use of antibiotics and those identified more recently (Fig 6C). This shift from bacteriocin production to antimicrobial resistance is presumably due to the high selective pressure undergone within bacterial population for antibiotic resistance, although it remains unclear why bacteriocins are now less frequent in ColE1 plasmids. The same ColE1 backbones are identified either as bacteriocin-producing ColE1, as antimicrobial resistant ColE1 or as cryptic. Indeed, most ColE1 plasmids do encode neither bacteriocins nor AMR. This means that the functional shift may not be mediated by direct replacement of bacteriocin-producing loci by antimicrobial resistant genes, but by a genetic turnover of functions that currently tends to increase the frequency of AMR genes because of natural selection for this trait. Interestingly, the acquisition of AMR genes was concomitant with an increase in genes encoding transposases (Fig 6C), which may have facilitated their acquisition by ColE1 plasmids. Overall, this analysis supports early works, in which it was suggested that the acquisition of AMR genes after the antibiotic use was mediated by the same plasmids from the pre-antibiotic era [62].

Our results also raise intriguing and challenging questions that could be the aim of future research lines. (*i*) Are ColE1 plasmids present in other Orders of Proteobacteria? Our analysis revealed that the only plasmid already identified in *Pseudomonadales* [13] was phylogenetically distant from the remaining replicons. This suggests that ColE1 plasmids might be circulating within *Pseudomonadales* in underrepresented genera or in minor prevalence. Indeed, due to the divergent evolution of ColE1 replicons and the source of our HMM profiles, the circulation of distant variants of ColE1 cannot be discarded. (*ii*) What are the consequences of the frequent occurrence of *repB* in ColE1 plasmids from *Aeromonadales*? The extended co-occurrence of both the ColE1 *ori* and *repB* suggests that rather than switching the plasmid’s mechanism of replication, there might be a synergistic effect between both, making these replicons more successful within this Order. (*iii*) Is the ColE1 *ori* functional when the replicon co-integrates with large plasmids? If the ColE1 *ori* increases the plasmid copy number (PCN) of the large one, it could make the co-integrate unstable due to its higher fitness cost. This could have happened after the integration of the ColE1 plasmid pIP843 with an additional replicon in the co-integrate pE66An [36], where the ColE1 *ori* was truncated after the recombination event. In contrast, if the PCN is not modified, the genesis of ColE1 co-integrates could increase mobility via conduction, but also affect gene expression, fitness cost, and evolvability of the multicopy plasmid.

The frequent co-integration of ColE1 plasmids with additional replicons within *Enterobacterales* suggests they may have a more determinant role in the evolution of the bacterial plasmidome than previously envisaged. The shift in their cargo genes from bacteriocin production to antimicrobial resistance suggests these plasmids are becoming important drivers of the spread of antibiotic resistance.

## Materials and methods

### Collection of ColE1 plasmids characterized in the literature

To create a collection of ColE1 plasmids, we first looked for the ColE1 replicons that had been already described in the literature, examining every published work deposited in Pubmed (last accessed in July, 2020) using as query “ColE1”. We only retrieved those plasmids whose ColE1 origin of replication had been annotated, either the RNAI or the RNA II, obtaining a total number of 74 ColE1 plasmids (S1 Table). While examining the bibliography, it was noticed that ColE1 plasmids had been described in the Order *Pasteurellales* but, to date, no work has characterized their origin of replication. Therefore, we selected from literature and characterized 8 putative ColE1 plasmids described in *Pasteurellales* to include ColE1 replicons from this taxon in our analysis. This way, our initial ColE1 collection was constituted by 81 plasmids, representing our reference dataset (S1 Table).

### Characterization of ColE1 replicons from *Pasteurellales*

The eight plasmids selected for the description of their ColE1 origin of replication in *Pasteurellales* were pB1000 (DQ840517), pIG1 (NC_001774.1), pLS88 (L23118), pAB2 (Z21724), pB1002 (JQ773456), pB1005 (NC_012215.1), pB1006 (NC_012216.1) and pB1000’ (NC_019177.1). To characterize their origin of replication we followed different approaches: *i*) the current literature available on their origin of replication; *ii*) multiple sequence alignments of the origin of replication among the different plasmids; *iii*) data from an RNA-Seq analysis of *H*. *influenzae* RdKW20 carrying pB1000 available in the European Nucleotide Archive under the Accession Number PRJEB44283; and *iv*) an *in silico* analysis of the secondary structure of the putative ColE1 RNAs (S14 Fig). Detailed information on this analysis is available in the S1 Text.

Still, to validate our results: *i*) we corroborated that the elements of the ColE1 origin of replication were conserved among the 8 plasmids; *ii*) we demonstrated that ColE1 plasmids that have been described coexisting within a cell show key mutations in their RNAs allowing their compatibility [19,24]; and *iii*) we verified that mutations in specific nucleotides of the RNAs modify the plasmid copy number [63], as it has been demonstrated in ColE1 plasmids from *Enterobacterales* [9] (S1 Text and S15 Fig).

### Construction of HMM profiles for ColE1 plasmids

For the construction of Hidden Markov Model (HMM) profiles, we first performed multiple sequence alignments (MSA) of the 81 ColE1 plasmids collected in our reference dataset using MAFFT [64], version 7.450, options—*globalpair* and—*maxiterate 1000* and examined the results with Geneious Prime (2019.0.4) for the detection of artifacts. We performed two different MSAs, the first one was specific for the RNAI sequence of the ColE1 plasmids, obtained from their respective published works (S1 Table). The second MSA was broader, including the whole *ori* region, between the RNAII promoter and the *oriV* site, thus including both RNAs and their promoters.

Once we had the alignments, we used HMMer (http://hmmer.org, last accessed December 2020), version 3.1b2 and built the HMM profiles with hmmbuild. Due to the remarkable sequence disagreements between ColE1 plasmids from different Orders, we constructed specific profiles for each Order of bacteria in addition to a profile including all the ColE1 plasmids. All the HMM profiles were used in our ColE1 search in order to increase our sensitivity.

### Identification of ColE1 plasmids

For the identification of ColE1 plasmids, we used the dataset of complete bacterial genomes from NCBI RefSeq (last accessed in September, 2019). We retrieved the 20,523 plasmids following the classification of the replicon as “plasmid” or “chromosome” within the GenBank file. For the detection of ColE1 plasmids, we combined the search for the aforementioned HMM profiles using the HMMer tool hmmsearch with a parallel search using PlasmidFinder [26]. To increase the specificity of our search, only those plasmids identified with both HMM profiles (*ori* and RNAI) were retrieved for further analysis. When using the RNAI HMM profile, the E-value threshold was augmented to 0.01 due to its short sequence (~100 bp) following the recommendations of the authors, whereas in the complete *ori* HMM profile, the E-value threshold was maintained at the default 0.001.

The 1,056 plasmids identified during the search were examined using Geneious Prime (2019.0.4) to verify their ColE1 origin of replication (presence of both the RNA I, RNA II and *oriV* site). After this inspection, 53 ColE1 replicons were discarded from different reasons: 15 sequences were incomplete plasmids with a ColE1 *ori* partially sequenced, 17 were actual cloning vectors and 21, although identified during the search, did not show the characteristic ColE1 origin of replication when manually inspected. To the 1,003 remaining plasmids, we added 32 additional replicons employed for the construction of the HMM profiles and absent within RefSeq, reaching 1,035 plasmids. These 32 elements obtained from the literature, but absent from RefSeq, were not used to estimate the ColE1 frequency within genera. All the plasmids were characterized according to the Inc/rep typing and the MOB typing, respectively. For the Inc/rep typing we used PlasmidFinder [26], version 2.0.1, with a minimum identity threshold of 95% and a minimum coverage threshold of 60%, with both the *Enterobacteriaceae* and Gram positive databases (last update on January 1^st^, 2021). We considered the following results as ColE1-like representatives: ColRNAI, Col(pHAD28), Col(YF27601), Col440I and Col440II. For the MOB-typing we used the online version of MOBscan [65] (last accessed on January, 2021) with the default parameters, which employed the program hmmscan, version 3.3, and the MOBfamDB database. Detailed information on the plasmids is available in the S2 Table.

### Functional analysis of ColE1 plasmids

Among the 1,035 ColE1 plasmids– 1,003 identified in RefSeq plus 32 additional plasmids described in the literature but absent from RefSeq–only the 958 circular sequences were considered for the analysis of plasmid size. Among them, just the 889 canonical ColE1 plasmids (<25kb) were further used for the functional analysis. The sequences were annotated using Prokka, version 1.13 [66], and the results were manually curated using Geneious Prime(2019.0.4). The resulting genes were classified in 6 groups and 27 subgroups attending to their functions and frequency (S4 Table). As certain sections of this work focus on specific functions of these plasmids such as antimicrobial resistance and bacteriocin production, we further validated the antimicrobial resistance genes using ResFinder version 4.0 [67], with a minimum identity threshold of 90% and a minimum coverage threshold of 60%. The genes encoding bacteriocins identified with Prokka were further validated with the online tool blastx (https://blast.ncbi.nlm.nih.gov/Blast.cgi, last accessed December 2020), annotating the bacteriocin according to the best match from the RefSeq database of Reference Proteins.

### Phylogenetic analysis

The phylogenetic analysis of the 1,035 ColE1 plasmids was performed from a MSA of the ColE1 origin of replication (*ori*) region, defined as the region between the RNA II promoter to the *oriV* site, using MAFFT [64], version 7.450, options—*globalpair* and—*maxiterate 1000* and examined the result with Geneious Prime (2019.0.4). The phylogenetic tree was inferred by maximum-likelihood using IQ-Tree [68], version 1.6.1, with 1000 ultrafast bootstrap experiments [69] and the ModelFinder function [70], being the selected model indicated in the legend of each figure. The visualization of the inferred tree was performed with iTOL [71], version 5.7. Additional phylogenetic analyses were performed in this work focusing on the relaxase protein (n = 339), the ColE1 origin of replication within large plasmids in *Enterobacterales* (n = 61) and the ColE1 *ori* and RepB protein within *Aeromonadales* (n = 29). All these analyses followed the same procedure described for the 1,035 ColE1 origins of replication. The Software Dendroscope [72], version 3.7.3, was used for the tanglegram analysis of both the ColE1 *ori* and RepB phylogenetic trees and its visualization. All the phylogenetic trees in Newick format have been included in the supplementary material (S4 Text).

### Identification of ColE1/RepB plasmids in *Aeromonadales*

The identification of further ColE1 plasmids within *Aeromonadales*, as well as plasmids encoding the *repB* gene described in pAsa10 (NZ_ MF621616.1) and p2_045096 (NZ_CP028563.1), was performed within the Assemblies database of NCBI RefSeq (accessed November, 2020), retrieving the 515 entries belonging to *Aeromonadales*. First, for the identification of ColE1 plasmids we used the aforementioned HMM profiles specific for *Aeromonadales*, with the HMMer tool hmmsearch, following the same procedure as previously described. Among the 217 sequences harboring a ColE1 origin of replication, only the 40 circular ones were selected for further analysis: 36 plasmids (*μ* = 9,36 kb) and 4 chromosomes (*μ* = 4,88 Mb).

Among the latter 40 sequences, 16 encoded an homolog to pAsa10 and p2_045096 *repB*. Therefore, we used the 16 *repB* genes for the construction of a new HMM profile, performing an MSA using MAFFT, options—*maxiterate 1000* and—*global-pair*. The MSA was visualized with Geneious Prime (2019.0.4). Then, the RepB HMM profile was built with the HMMer tool hmmbuild and used for the identification of the gene within the same RefSeq database, using the HMMer tool hmmsearch (default, E-value < 0.001). Among the 467 sequences with the gene, only the 63 circular were selected for the analysis: 60 plasmids (*μ* = 43,24 kb) and 3 chromosomes (*μ* = 4,90 Mb). A total of 28 sequences were identified in the searches for both ColE1 *ori* and *repB*.

### Identification of ColE1 plasmids from the Murray Collection

Raw Illumina Sequencing data from 370 isolates of the Murray Collection was downloaded from the European Nucleotide Archive, available under the accession number PRJEB3255 [61]. We performed a quality control using the software FastQC [73], version 0.11.9, and trimmed the reads using fastp [74], version 0.20.1. Putative plasmids were assembled from the Illumina reads using PlasmidSPAdes [75], version 3.15.2, with the default parameters. We obtained a total number of 40,138 contigs, with an average size of 2,243 bp. Then, we used our *Enterobacterales* ColE1 HMM profile and PlasmidFinder to identify ColE1 replicons within the contigs, following the same conditions as previously specified. A total number of 173 sequences were retrieved although 58 were afterwards discarded due to various reasons (S5 Table): 18 were ColE1 replicons that presented partially sequenced the *ori*, 6 were too short sequences (*μ* = 413,2 bp) and 34 were not actual ColE1 replicons after manual inspection. The functional contribution of the 115 ColE1 plasmids (*μ* = 8,667.6 bp) identified was analyzed using Prokka and ResFinder, with the aforementioned parameters (S6 Table).

### Statistics and data visualization

The different statistical tests used during this work (ANOVA, Student t-test, correlation test, Chi-squared test, Fisher’s exact test) were performed with the default R package *stats* in RStudio, version 3.6.1. Most data visualization was performed with the R package *ggplot2* with few aforementioned exceptions. Plasmid representations were drawn with Easyfig [76], version 2.2.5.

## Supporting information

S1 Text Characterization of the ColE1 origin of replication in *Pasteurellales*. (DOCX)

S2 Text ColE1 replicons identified with Rep proteins in *Pasteurellales*. (DOCX)

S3 Text ColE1 co-integrates in *Enterobacterales*. (DOCX)

S4 Text Phylogenetic trees represented in the work in Newick format. (TXT)

S1 Table ColE1 plasmids described in the literature. Collection of plasmids already described in the literature and used for building the HMM profiles.
(XLSX)

S2 Table Collection of 1,035 ColE1 plasmids. Collection of 1,035 ColE1 plasmids identified in RefSeq with both the HMM profiles and PlasmidFinder.
(XLSX)

S3 Table Collection of ColE1/RepB plasmids from *Aeromonadales*. Collection of plasmids identified in *Aeromonadales* encoding a ColE1 *ori* and/or *repB*.
(XLSX)

S4 Table Functional contribution of ColE1 plasmids. Annotation of the complete canonical ColE1 plasmids (<25kb) from the 1,035 ColE1 collection.
(XLSX)

S5 Table ColE1 plasmids from the Murray Collection. ColE1 replicons identified within the ENA project PRJEB44283.
(XLSX)

S6 Table Functional contribution of ColE1 plasmids from the Murray Collection. Annotation of the ColE1 plasmids identified within the ENA project PRJEB44283.
(XLSX)

S1 Fig Phylogenetic tree of the ColE1 plasmids from *Aeromonadales*. Phylogenetic tree of the 12 ColE1 origins of replication identified in *Aeromonadales*. The colors of the first column represent the Genus in which the replicon was identified, the second column represents the Species and the third column indicates the presence or absence of a relaxase. The legend is at the right of the figure. The bootstrap values are indicated with a number next to each node. The phylogenetic tree was inferred following the best-fit model, K2P+G4.
(TIFF)

S2 Fig Phylogenetic tree of the ColE1 plasmids from *Vibrionales*. Phylogenetic tree of the 20 ColE1 origins of replication identified in *Vibrionales*. The colors of the first column represent the Genus in which the replicon was identified, the second column represents the Species and the third column indicates the presence or absence of a relaxase. The legend is at the right of the figure. The bootstrap values are indicated with a number next to each node. The phylogenetic tree was inferred following the best-fit model, TIM3e+G4.
(TIFF)

S3 Fig Phylogenetic tree of the ColE1 plasmids from *Pasteurellales*. Phylogenetic tree of the 38 ColE1 origins of replication identified in *Pasteurellales*. The colors of the first column represent the Genus in which the replicon was identified, the second column represents the Species and the third column indicates the presence or absence of a relaxase, either MOBV or MOBP. The legend is at the right of the figure. The bootstrap values are indicated with a number next to each node. The phylogenetic tree was inferred following the best-fit model, HKY+F+G4.
(TIFF)

S4 Fig Phylogenetic tree of the ColE1 plasmids from *Enterobacterales*. Phylogenetic tree of the 964 ColE1 origins of replication identified in *Enterobacterales*. The colors of the first column represent the Genus in which the replicon was identified, the second column represents the Species and the third column indicates the presence or absence of a relaxase, either MOBP, MOBQ or truncated (ΔMOB). The legend is at the right of the figure. The bootstrap values are indicated with the nodes and branch colors. Bootstraps under 50 are represented in black, whereas bootstraps over 50 follow the legend at the right. The phylogenetic tree was inferred following the best-fit model, SYM+R7.
(TIFF)

S5 Fig Phylogenetic tree of the ColE1 *ori* of ColE1-only and ColE1/RepB plasmids from *Aeromonadales*. The colors of the column at the right of the tree represent if the plasmid is ColE1-only or ColE1/RepB. The legend is at the right of the figure. The bootstrap values are indicated with a number next to each node. The phylogenetic tree was inferred following the best-fit model, TVMe+R3.
(TIFF)

S6 Fig Location of the ColE1 *ori* and *repB* gene within the ColE1/RepB plasmids from *Aeromonadales*. At the left, it is represented the phylogenetic tree of the ColE1 *ori* from the ColE1/RepB plasmids, as indicated in Fig 3. At the end of every branch it is indicated the host Species (colored square, legend at the bottom), the Accession Number and the plasmid size. At the right of the figure, it is represented the genetic content of the ColE1/RepB plasmids. The ColE1 *ori* is represented with a purple rectangle whereas *repB* with a purple arrow. The remaining genes are represented with colored arrows, being the legend at the bottom of the figure.
(TIFF)

S7 Fig Comparison between the phylogeny of the ColE1 *ori* and gene *repB*. Comparison of the phylogenetic trees of the ColE1 origin of replication (left) (best-fit model JTT+G4) and the gene *repB* (right) (TN+F+I+G4). The color of the branches represents the comparison metric. The legend is shown at the bottom of the figure. A score of 1 denotes the subtree structure of the node is identical to the subtree structure of its best corresponding node. The figure was performed with the phylo.io tool [77].
(TIFF)

S8 Fig ColE1 co-integrate in *Vibrio campbellii*. Schematic representation of the ColE1 co-integrate identified in *V*. *campbellii* (NZ_CP026317.1). The complete plasmid (194 kb) is represented at the top of the figure. The purple square indicates the genetic environment of the ColE1 origin of replication, which is represented at the bottom of the figure. The ColE1 *ori* and remaining genes are represented with colored squares and arrows, being the legend at the bottom of the figure.
(TIFF)

S9 Fig ColE1 co-integrates from *Enterobacterales*. Schematic representation of the genetic environment of the ColE1 *ori* in the most-represented clades of ColE1 co-integrates from *Enterobacterales* (Fig 4). The ColE1 *ori* and remaining genes are represented with colored squares and arrows, being the legend at the top of the figure. The Accession Number and size of each plasmid is indicated at the middle of the figure.
(TIFF)

S10 Fig Phylogenetic tree of the relaxase from *Aeromonadales*. Phylogenetic tree of the ColE1 relaxases from *Aeromonadales*. The colors of the first column represent the Genus and the second column represents the Species. The legend is at the right of the figure. The bootstrap values are indicated with a number next to each node. The phylogenetic tree was inferred following the best-fit model, JTT+F+G4.
(TIFF)

S11 Fig Phylogenetic tree of the relaxase from *Pasteurellales*. Phylogenetic tree of the ColE1 relaxases from *Pasteurellales*. The colors of the first column represent the Genus and the second column represents the Species. The legend is at the right of the figure. The bootstrap values are indicated with a number next to each node. The phylogenetic tree was inferred following the best-fit model, VT+G4.
(TIFF)

S12 Fig Phylogenetic tree of the relaxase from *Enterobacterales*. Phylogenetic tree of the ColE1 relaxases from *Enterobacterales*. The colors of the first column represent the Genus, the second column represents the presence or absence of *rop* and the third one indicates the PlasmidFinder result. The legend is at the right of the figure. The phylogenetic tree was inferred following the best-fit model, JTT+F+I+G4.
(TIFF)

S13 Fig Phylogenetic tree of the ColE1 *ori* from *Enterobacterales* including the plasmids from the Murray Collection. The colors of the column represent the origin of the plasmid, Murray Collection or RefSeq. The legend is at the right of the figure. The phylogenetic tree was inferred following the best-fit model, SYM+R8.
(TIFF)

S14 Fig Putative RNA I of the ColE1 plasmids from *Pasteurellales*. Schematic representation of the secondary structure of the putative RNA I from eight ColE1 plasmids from *Pasteurellales*. The name and Accession Number of each plasmid is indicated at the top of each sequence. The color of the nucleotides indicates the base-pair probabilities, from 0 to 1, being the legend next to each sequence. The secondary structure and probabilities were inferred with RNAfold WebServer.
(TIFF)

S15 Fig Putative RNA I of coexisting ColE1 plasmids from *Pasteurellales*. Schematic representation of the secondary structure of the putative RNA I from different ColE1 plasmids from *Pasteurellales* described coexisting within the cell. The different plasmid combinations are represented separated in the boxes A, B, C and D, being indicated the Species isolate, the plasmid names and the published reference. The Accession Number of each plasmid is indicated at the top of the sequence. The letters and arrows show the dissimilarities identified among the coexisting plasmids, in black those affecting the loop and in grey those affecting the stem. The color of the nucleotides indicates the base-pair probabilities, from 0 to 1, being the legend at the top right of each box. The secondary structure and probabilities were inferred with RNAfold WebServer.
(TIFF)
