# Supplementary material for: Evolution of ColE1-like plasmids across γ-Proteobacteria: From bacteriocin production to antimicrobial resistance
Source: PLoS Genet. 2021 Nov 30;17(11):e1009919. doi: 10.1371/journal.pgen.1009919 (PMC8683028; doi:10.1371/journal.pgen.1009919)
Supplement: S1 Text — (DOCX) [file pgen.1009919.s001.docx]

**S1 Text. Characterization of the ColE1 origin of replication in *Pasteurellales***

As mentioned in the main text (see Materials and methods), ColE1-like plasmids within *Pasteurellales* have been previously described [1–3], although their ColE1 origin of replication is still uncharacterized. To include ColE1 replicons from this taxa into our ColE1 HMM profiles, we characterized the origin of replication of 8 putative ColE1 plasmids previously described in *Pasteurellaceae*: pLS88 (L23118.1) [4], pAB2 (Z21724.1) [5], pIG1 (NC_001774.1) [6], pB1000 (DQ840517.1) [1], pB1002 (JQ773456.1) [2], pB1005 (NC_012215.1) [2], pB1006 (NC_012216.1) [2] and pB1000’ (NC_019177.1) [7].

Willson and collaborators carried out the first experimental work analyzing the origin of replication of one of these plasmids, locating the origin of replication of pLS88 in a region of ~800 bp with no proteins encoded [4]. It is encoded approximately half kilobase upstream the relaxase module, in the same location where the ColE1 plasmids from *Enterobacterales* show their origin of replication [8]. To test the transcription within this area, we analyzed an already performed RNA-Seq of *Haemophilus influenzae* RdKW20 carrying pB1000, available in the European Nucleotide Archive under the Accession Number PRJEB44283. Although the RNA-Seq data was not clean enough to accurately identify the transcripts from the putative origin of replication of pB1000, there was a large number of reads within this region, demonstrating its high transcription activity despite the lack of proteins encoded.

To identify the RNAs I and II, we examined *in silico* the origin of replication of the eight plasmids. Our analysis revealed a region conserved among all the plasmids that could correspond to the sequence of the RNA I transcript. This putative RNA I showed an average length of 99.4 bp and a 91.4% pairwise identity among the eight plasmids. As in the case of ColE1 plasmids from *Vibrionales*, the putative RNA I showed an stable secondary structure of two stem loops [9], represented in the S13 Figure. In contrast, inferring the complete length of the RNA II transcript is particularly challenging without experimental work. However, in ColE1 plasmids from *Enterobacterales*, *Aeromonadales* and *Vibrionales*, the RNA II transcript usually starts at the end of the RNA I, in the complementary strand. Indeed, in the eight ColE1 plasmids analyzed, we manually inspected the sequences and identified a conserved promoter with the -35 box TTGACA located 36bp (pLS88, pIG1, pB1005, pB1006) and 37bp (in pAB2, pB1000, pB1000’ and pB1002) upstream the end of the RNA I transcript, suggesting the same organization of both RNAs. In this work, we set the complete origin of replication from the RNA II promoter to the *oriV* site, approximately 500 bp downstream the RNA II promoter. We manually examined the sequence the eight plasmids from *Pasteurellales*, and identified a putative *oriV* site, 512-515bp upstream the -35 box promoter of the RNA II, showing the same characteristics than the *oriV* of ColE1 plasmids from *Enterobacterales* 20bp downstream a poli-GC sequence.

To validate our results, we first verified that all the genetic features were conserved among the 8 ColE1 plasmids: the RNA II promoter, the RNA I sequence with the distinctive secondary structure of two stem loops and the putative *oriV* site. Secondly, it has been demonstrated that single nucleotide polymorphisms (SNPs) within the stem loops of the RNA I and II can prompt compatibility between different ColE1 plasmids [10]. Indeed, we have previously identified some of the aforementioned ColE1 plasmids coexisting within *Pasteurellaceae* wild-type isolates, as well as with additional ColE1 replicons such as pB1002, pB1003 or p9956 [2]. Hence, we explored if mutations within the putative RNAs were involved in their compatibility. In all the combinations, we identified SNPs in the stem loops of their putative RNAs, thus, supporting our *in silico* results (S14 Fig). Additionally, in other published work, we identified a SNP in the plasmid pB1000 that confer compatibility to two different alleles of the same replicon [3]. In this work, we corroborated that the mutation identified was indeed affecting one of the stem loops of the putative RNAs (S14 Fig).

Lastly, it is known that SNPs in the stem loops of the RNAs might modify their plasmid copy number [10]. In a previous work using the plasmid pB1000, we demonstrated that mutations within specific nucleotides of the *ori* affecting the RNAs and the RNA II promoter, did affect the plasmid copy number, and in consequence, the stability of pB1000 within *Escherichia coli* DH5α [11]. The major analyzed mutation was the 3880C>A (pB1000, accession number DQ840517), nucleotide located in a stem loop of the RNA I, and produced a ten fold increase in the plasmid copy number [11].

All these findings indicate the accuracy of our ColE1 *ori* characterization in *Pasteurellales*, with the transcription of both an RNA I and RNA II complementary via two stem loops. Furthermore, as it has been demonstrated in *Enterobacterales*, we have demonstrated that SNPs within the origin of replication of these small plasmids are responsible for their compatibility within the cell and are able to trigger plasmid copy number modifications.

**Reference**

1. San Millan A, Escudero JA, Catalan A, et al. β-Lactam Resistance in Haemophilus parasuis Is Mediated by Plasmid pB1000 Bearing blaROB-1. *Antimicrob Agents Chemother*. 2007;51(6):2260-2264. doi:10.1128/AAC.00242-07

2. San Millan A, Escudero JA, Gutierrez B, et al. Multiresistance in Pasteurella multocida is mediated by coexistence of small plasmids. *Antimicrob Agents Chemother*. 2009;53(8):3399-3404. doi:10.1128/AAC.01522-08

3. Santos-Lopez A, Bernabe-Balas C, Ares-Arroyo M, et al. A Naturally Occurring Single Nucleotide Polymorphism in a Multicopy Plasmid Produces a Reversible Increase in Antibiotic Resistance. *Antimicrob Agents Chemother*. 2017;61(2). doi:10.1128/AAC.01735-16

4. Willson PJ, Albritton WL, Slaney L, Setlow JK. Characterization of a multiple antibiotic resistance plasmid from Haemophilus ducreyi. *Antimicrob Agents Chemother*. 1989;33(9):1627-1630. doi:10.1128/aac.33.9.1627

5. Wood AR, Lainson FA, Wright F, Baird GD, Donachie W. A native plasmid of Pasteurella haemolytica serotype A1: DNA sequence analysis and investigation of its potential as a vector. *Res Vet Sci*. 1995;58(2):163-168. doi:10.1016/0034-5288(95)90071-3

6. Wright CL, Strugnell RA, Hodgson AL. Characterization of a Pasteurella multocida plasmid and its use to express recombinant proteins in P. multocida. *Plasmid*. 1997;37(1):65-79. doi:10.1006/plas.1996.1276

7. San Millan A, Garcia-Cobos S, Escudero JA, et al. Haemophilus influenzae clinical isolates with plasmid pB1000 bearing blaROB-1: fitness cost and interspecies dissemination. *Antimicrob Agents Chemother*. 2010;54(4):1506-1511. doi:10.1128/AAC.01489-09

8. Ares-Arroyo M, Bernabe-Balas C, Santos-Lopez A, et al. PCR-Based Analysis of ColE1 Plasmids in Clinical Isolates and Metagenomic Samples Reveals Their Importance as Gene Capture Platforms. *Front Microbiol*. 2018;9:469. doi:10.3389/fmicb.2018.00469

9. Le Roux F, Davis BM, Waldor MK. Conserved small RNAs govern replication and incompatibility of a diverse new plasmid family from marine bacteria. *Nucleic Acids Res*. 2011;39(3):1004-1013. doi:10.1093/nar/gkq852

10. Camps M. Modulation of ColE1-like Plasmid Replication for Recombinant Gene Expression. *Recent Pat DNA Gene Seq*. 2010;4(1):58-73.

11. Bernabe-Balas C. *Identificación y Caracterización Molecular de Mecanismos de Adaptación Plasmídica a Nuevas Familias Bacterianas (Doctoral Dissertation)*. Universidad Complutense de Madrid; 2019.
