# Supplementary material for: Evolution of ColE1-like plasmids across γ-Proteobacteria: From bacteriocin production to antimicrobial resistance
Source: PLoS Genet. 2021 Nov 30;17(11):e1009919. doi: 10.1371/journal.pgen.1009919 (PMC8683028; doi:10.1371/journal.pgen.1009919)
Supplement: S2 Text — (DOCX) [file pgen.1009919.s002.docx]

**S2 Text. ColE1 replicons identified with Rep proteins in *Pasteurellales***

As mentioned in the main text (see Results), among the 1,035 ColE1 replicons identified, we observed three small (<25 kb) ColE1 plasmids from *Pasteurellales* encoding a Rep protein in addition to the ColE1 origin of replication. Although the sequence of the three ColE1/Rep plasmids has been previously analyzed, their ColE1 origin of replication has not been detected [1–3].

First, the multiresistance plasmid pOV (NC_019381) was recently described in *Pasteurella multocida*, typed as IncX and proven to be stably maintained in *E. coli* [2]. In our ColE1 identification, we observed the characteristic ColE1 *ori* from *Pasteurellales* encoded upstream *mobC* and 565bp downstream the gene *repX*. Thus, pOV is actually a ColE1/IncX plasmid, which explains its exceptional host range, stable in *E. coli* due to its IncX replication and in *P. multocida* due to its *Pasteurellales* ColE1 *ori*.

Secondly, the two multiresistance plasmids pKMA1467 (NC_007096) and pKMA202 (NC_009624) were described in *Actinobacillus porcitonsillarum* carrying a *repB*/*repA* operon [1]. Matter and collaborators did mention that in pKM1467 there was a region similar to the putative origin of replication of pLS88 (L23118.1) and pKMA756 (NC_007097.1). In this work, we have identified within this region – in both pKMA1467, pLS88 and pKMA756 – a *Pasteurellales* ColE1 origin of replication, demonstrating that pKMA1467 not only encodes for the *repB*/*repA* genes, but also for a ColE1 origin of replication. As the same *repA* gene was identified in other small non-ColE1 plasmids from *Pasteurellaceae* (pJR1, NC_004771.1; pMVSCS1, NC_003411.1; p250, AY300023.1) and *Neisseriaceae* (pFA3, DQ355980.1), we demonstrated that both pKMA1467 and pKMA202 are actually chimeric plasmids encoding for both origin of replications, and furthermore, confirmed that recombination events between small plasmids is a common phenomenon in *Pasteurellales*.

**Reference**

1. Matter D, Rossano A, Sieber S, Perreten V. Small multidrug resistance plasmids in Actinobacillus porcitonsillarum. *Plasmid*. 2008;59(2):144-152. doi:10.1016/j.plasmid.2007.11.003

2. López-Ochoa AJ, Sánchez-Alonso P, Vázquez-Cruz C, et al. Molecular and genetic characterization of the pOV plasmid from Pasteurella multocida and construction of an integration vector for Gallibacterium anatis. *Plasmid*. 2019;103:45-52. doi:10.1016/j.plasmid.2019.04.003

3. Li Y, da Silva GC, Li Y, et al. Evidence of Illegitimate Recombination Between Two Pasteurellaceae Plasmids Resulting in a Novel Multi-Resistance Replicon, pM3362MDR, in Actinobacillus pleuropneumoniae. *Front Microbiol*. 2018;9:2489. doi:10.3389/fmicb.2018.02489
