# Supplementary material for: Evolution of ColE1-like plasmids across γ-Proteobacteria: From bacteriocin production to antimicrobial resistance
Source: PLoS Genet. 2021 Nov 30;17(11):e1009919. doi: 10.1371/journal.pgen.1009919 (PMC8683028; doi:10.1371/journal.pgen.1009919)
Supplement: S3 Text — (DOCX) [file pgen.1009919.s003.docx]

**S3 Text. ColE1 co-integrates in *Enterobacterales***

Here we present detailed information on the most relevant ColE1 co-integrates in *Enterobacterales* (see Results, Fig 4).

**Cluster A: ColE1/IncC plasmids**

In the phylogenetic tree of the ColE1 co-integrates (Fig 4), we observe a ColE1/IncC cluster of 8 plasmids from *K. pneumoniae*. The whole cluster is actually formed by three identical plasmids, sizing 187,611bp (NZ_CP024536.1, NZ_CP024550.1, NZ_CP024564.1), 217,685bp (NZ_ CP024557.1) and 221,606bp (NZ_CP024192, NZ_CP024522 and NZ_CP024529). The genetic environment of the ColE1 *ori* was identical among all of them (S9 Fig): a 14 kb sequence surrounded by two IS*4321* (IS*110* family) at each side and interrupting a mercury tolerance operon. In the region flanked by the transposases, there were genes responsible for tetracycline resistance (*tetA* and *tetR*).

The identical genetic environment surrounding the ColE1 *ori*, the highly conserved sequence among the complete plasmids and their limitation to *K. pneumoniae*, suggest that the co-integration of the ColE1 plasmid with the IncC replicon has recently occurred, but it has been stably maintained over time.

**Cluster B: ColE1/IncF and ColE1/NT plasmids**

The second group of plasmids is heterogenous, showing a high degree of dissimilarities in the genetic environment of the ColE1 origin of replication (S9 Fig) and suggesting different acquisitions of the ColE1 *ori* over time with no evolutionary history in common. Within the surroundings of the ColE1 *ori* it stands out the massive presence of transposases and a substantial number of antimicrobial resistance genes against diverse classes of antibiotics (S9 Fig). Within this cluster, there is a subgroup of five plasmids with a ColE1 *ori* extremely conserved (Fig 4, cluster B1), but showing different plasmid sizes (33,669bp - 279,104bp) and different hosts (both *Klebsiella* and *Escherichia*). Among these five replicons, it seems that the ColE1 *ori* is encoded within a region that has been moving as a unit surrounded by an IS*Ec9* and an IS*26*, including the mobilization of the carbapenemase CTX-M-14 together with the ColE1 *ori*.

**Cluster C: ColE1/IncN Plasmids**

We described a complex of 14 plasmids with 11 ColE1/IncN, 1 ColE1/IncN/IncF and 2 non-typeable replicons (Fig 4). Focusing on those plasmids classified as IncN with PlasmidFinder, we can split them into the recently described IncN2 and IncN3 families of plasmids (Fig 4). Nonetheless, in all the IncN2 and IncN3, the ColE1 origin of replication was identified in the same position, next to the conjugation machinery of these replicons (S9 Figure), denoting an extremely stable location for the ColE1 origin of replication within the plasmid even in different hosts (*Escherichia, Klebsiella, Citrobacter, Enterobacter*) and suggesting a successful relationship between both replicons. The four ColE1/IncN2 plasmids (cluster C1: NZ_CP034398, NC_019163, NC_024954, NC_015872) are the most relevant from a clinical point of view, already described mobilizing the carbapenemase NDM-1 among different genus of *Enterobacteriaceae* in different countries [1–3]. Though, this is the first time that the ColE1 *ori* has been identified, adjacent to the NDM-1-carrying Tn*5604* [1].

**Cluster D: ColE1/NT plasmids**

Lastly, there is a cluster of 14 plasmids (10 non-typeable, 3 ColE1/IncF and 1 ColE1 IncN), in which 12 out of the 14 show an extremely conserved ColE1 *ori* (Fig 4). In all the cases, the ColE1 *ori* has been co-integrated between a colicin genetic module (colicin itself, colicin immunity and colicin release) and a TraM recognition protein (putatively related to plasmid mobilization). In the 12 most conserved plasmids, the co-integration of the “colicin-ColE1 *ori*-mobilization” module locates next to a Tn*3* family transposon (S9 Fig). Despite the high similarity of the ColE1 *ori* and its genetic environment, these replicons are diverse in both size (from 25kb to 176kb) and host (both *Escherichia* and *Klebsiella*), which suggests that the co-integration of this module is a common phenomenon in the plasmidome of *Enterobacterales.* In many cases, the co-integration has been produced next to antimicrobial resistance genes, mainly against aminoglycosides and carbapenems (S9 Fig).

**Reference**

1. Poirel L, Bonnin RA, Nordmann P. Analysis of the Resistome of a Multidrug-Resistant NDM-1-Producing Escherichia coli Strain by High-Throughput Genome Sequencing. *Antimicrob Agents Chemother*. 2011;55(9):4224-4229. doi:10.1128/AAC.00165-11

2. Netikul T, Sidjabat HE, Paterson DL, et al. Characterization of an IncN2-type blaNDM-₁-carrying plasmid in Escherichia coli ST131 and Klebsiella pneumoniae ST11 and ST15 isolates in Thailand. *J Antimicrob Chemother*. 2014;69(11):3161-3163. doi:10.1093/jac/dku275

3. Chen Y-T, Lin A-C, Siu LK, Koh TH. Sequence of closely related plasmids encoding bla(NDM-1) in two unrelated Klebsiella pneumoniae isolates in Singapore. *PloS One*. 2012;7(11):e48737. doi:10.1371/journal.pone.0048737
