# Supplementary figures and images for: Evolution of ColE1-like plasmids across γ-Proteobacteria: From bacteriocin production to antimicrobial resistance
Source: PLoS Genet. 2021 Nov 30;17(11):e1009919. doi: 10.1371/journal.pgen.1009919 (PMC8683028; doi:10.1371/journal.pgen.1009919)

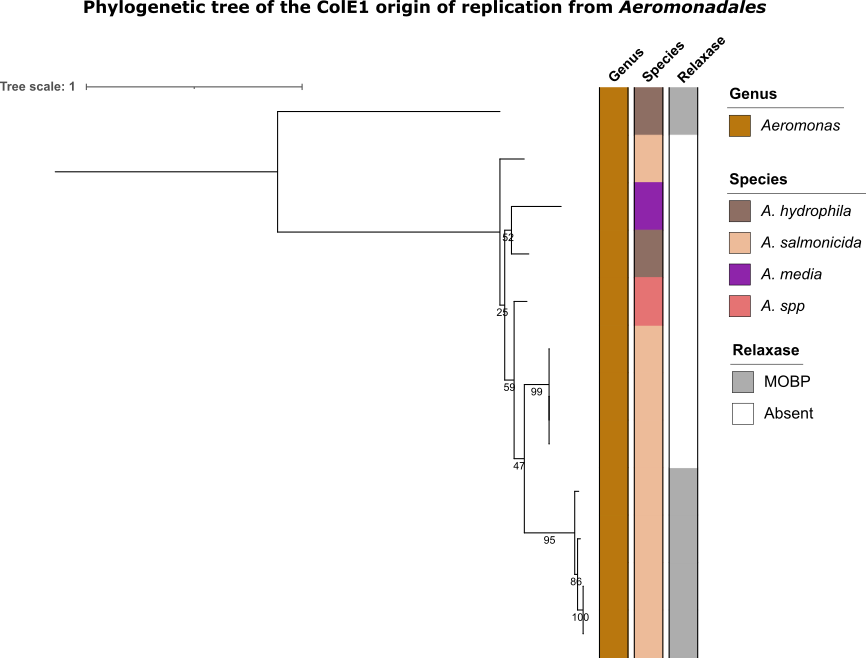

Supplement: S1 Fig — Phylogenetic tree of the 12 ColE1 origins of replication identified in Aeromonadales. The colors of the first column represent the Genus in which the replicon was identified, the second column represents the Species and the third column indicates the presence or absence of a relaxase. The legend is at the right of the figure. The bootstrap values are indicated with a number next to each node. The phylogenetic tree was inferred following the best-fit model, K2P+G4. (TIFF) [file pgen.1009919.s011.tiff]

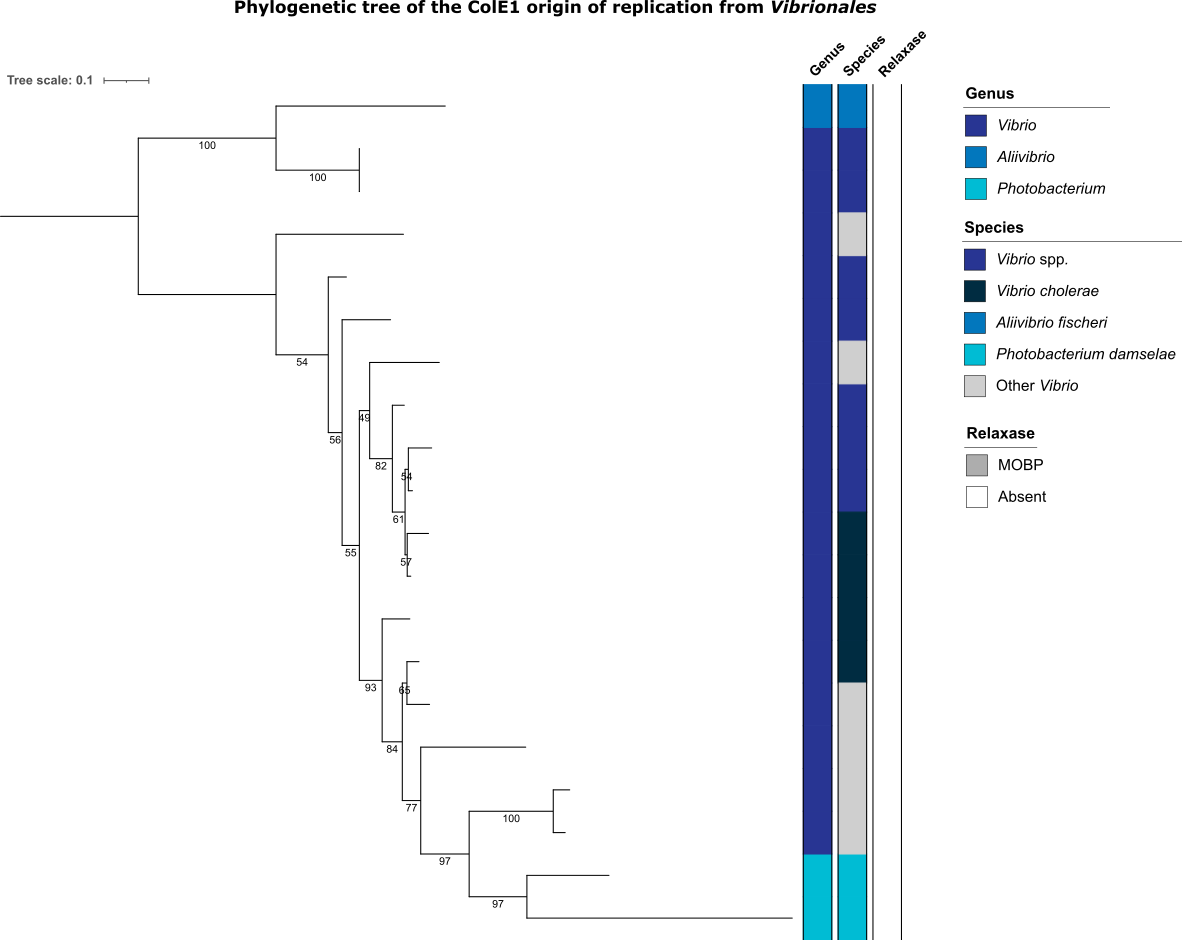

Supplement: S2 Fig — Phylogenetic tree of the 20 ColE1 origins of replication identified in Vibrionales. The colors of the first column represent the Genus in which the replicon was identified, the second column represents the Species and the third column indicates the presence or absence of a relaxase. The legend is at the right of the figure. The bootstrap values are indicated with a number next to each node. The phylogenetic tree was inferred following the best-fit model, TIM3e+G4. (TIFF) [file pgen.1009919.s012.tiff]

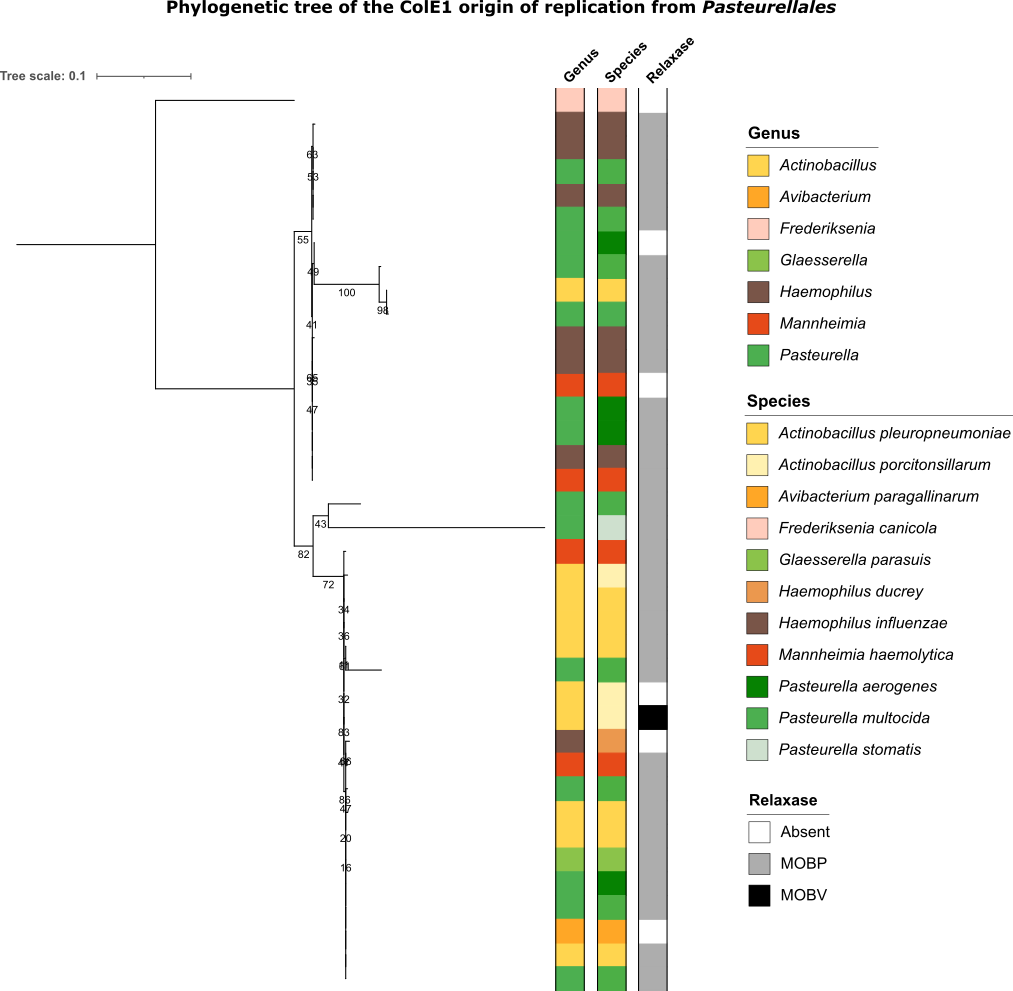

Supplement: S3 Fig — Phylogenetic tree of the 38 ColE1 origins of replication identified in Pasteurellales. The colors of the first column represent the Genus in which the replicon was identified, the second column represents the Species and the third column indicates the presence or absence of a relaxase, either MOBV or MOBP. The legend is at the right of the figure. The bootstrap values are indicated with a number next to each node. The phylogenetic tree was inferred following the best-fit model, HKY+F+G4. (TIFF) [file pgen.1009919.s013.tiff]

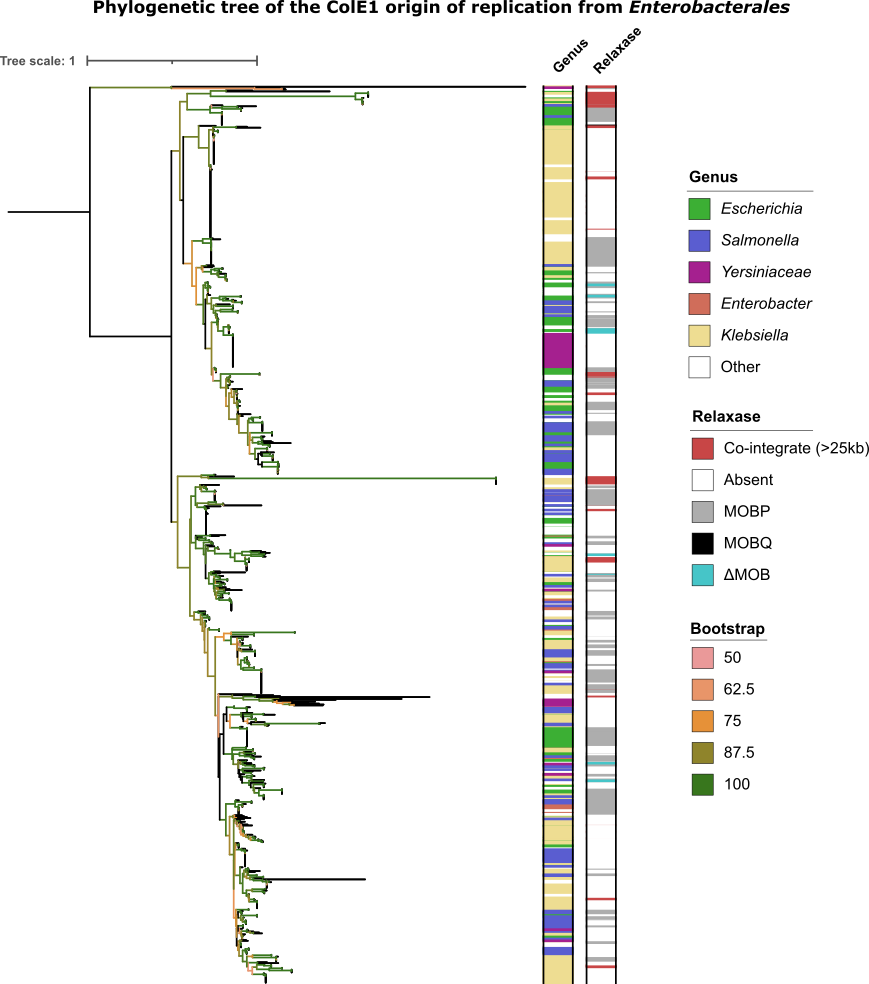

Supplement: S4 Fig — Phylogenetic tree of the 964 ColE1 origins of replication identified in Enterobacterales. The colors of the first column represent the Genus in which the replicon was identified, the second column represents the Species and the third column indicates the presence or absence of a relaxase, either MOBP, MOBQ or truncated (ΔMOB). The legend is at the right of the figure. The bootstrap values are indicated with the nodes and branch colors. Bootstraps under 50 are represented in black, whereas bootstraps over 50 follow the legend at the right. The phylogenetic tree was inferred following the best-fit model, SYM+R7. (TIFF) [file pgen.1009919.s014.tiff]

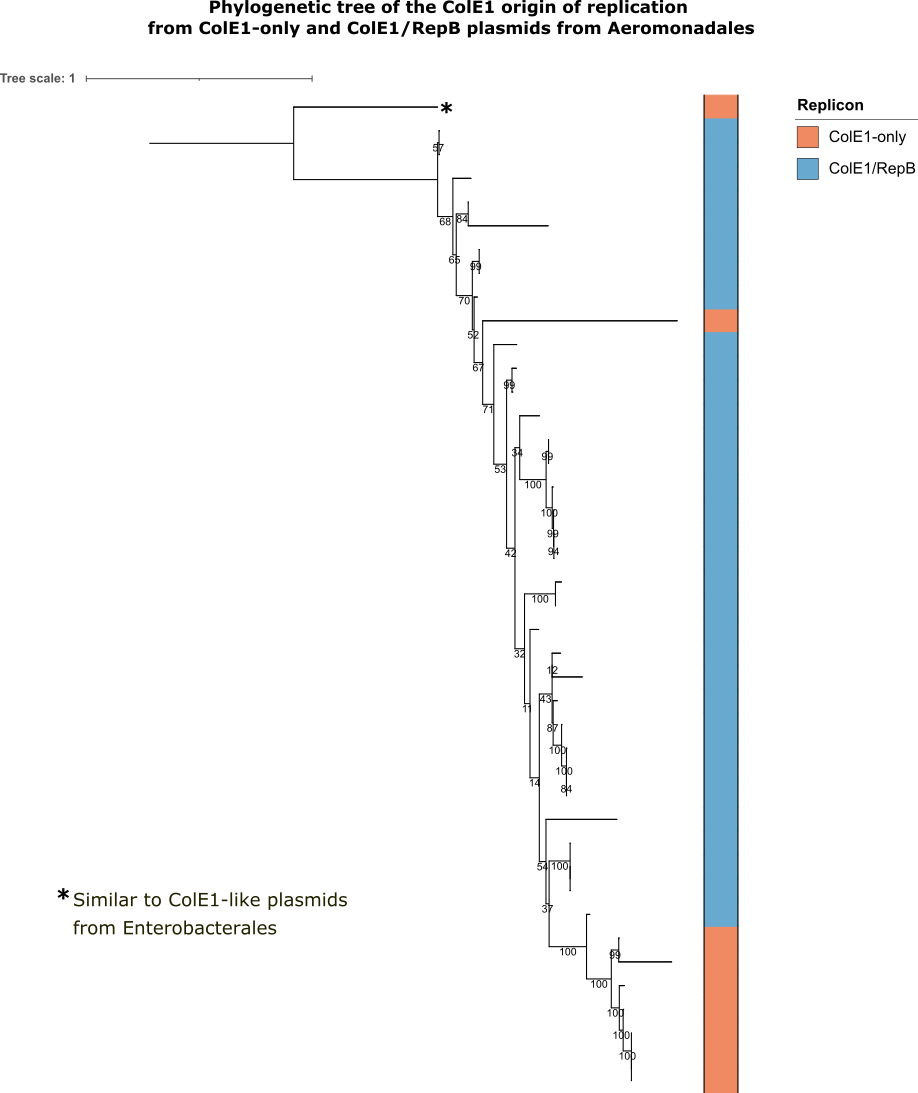

Supplement: S5 Fig — The colors of the column at the right of the tree represent if the plasmid is ColE1-only or ColE1/RepB. The legend is at the right of the figure. The bootstrap values are indicated with a number next to each node. The phylogenetic tree was inferred following the best-fit model, TVMe+R3. (TIFF) [file pgen.1009919.s015.tiff]

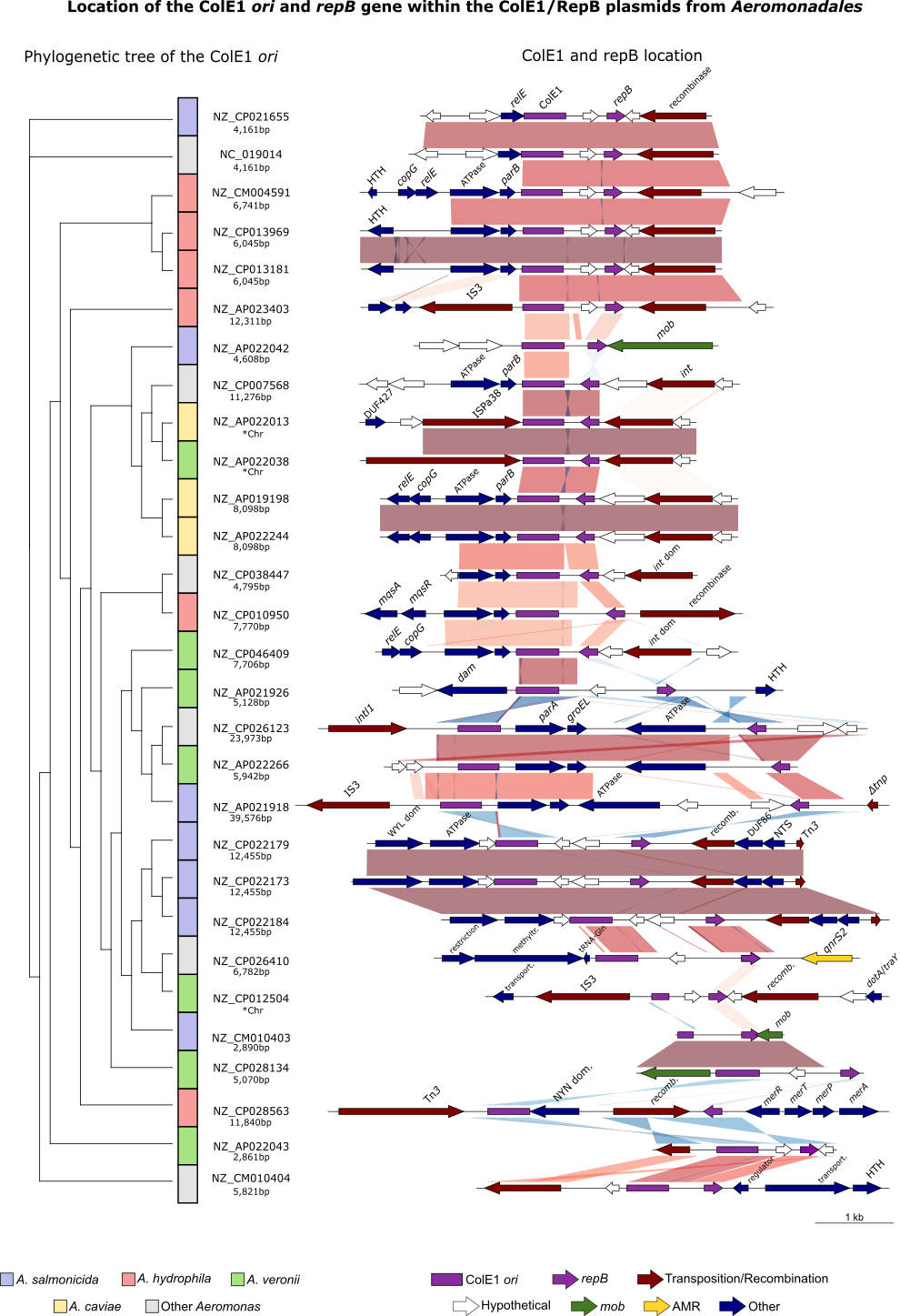

Supplement: S6 Fig — At the left, it is represented the phylogenetic tree of the ColE1 ori from the ColE1/RepB plasmids, as indicated in Fig 3. At the end of every branch it is indicated the host Species (colored square, legend at the bottom), the Accession Number and the plasmid size. At the right of the figure, it is represented the genetic content of the ColE1/RepB plasmids. The ColE1 ori is represented with a purple rectangle whereas repB with a purple arrow. The remaining genes are represented with colored arrows, being the legend at the bottom of the figure. (TIFF) [file pgen.1009919.s016.tiff]

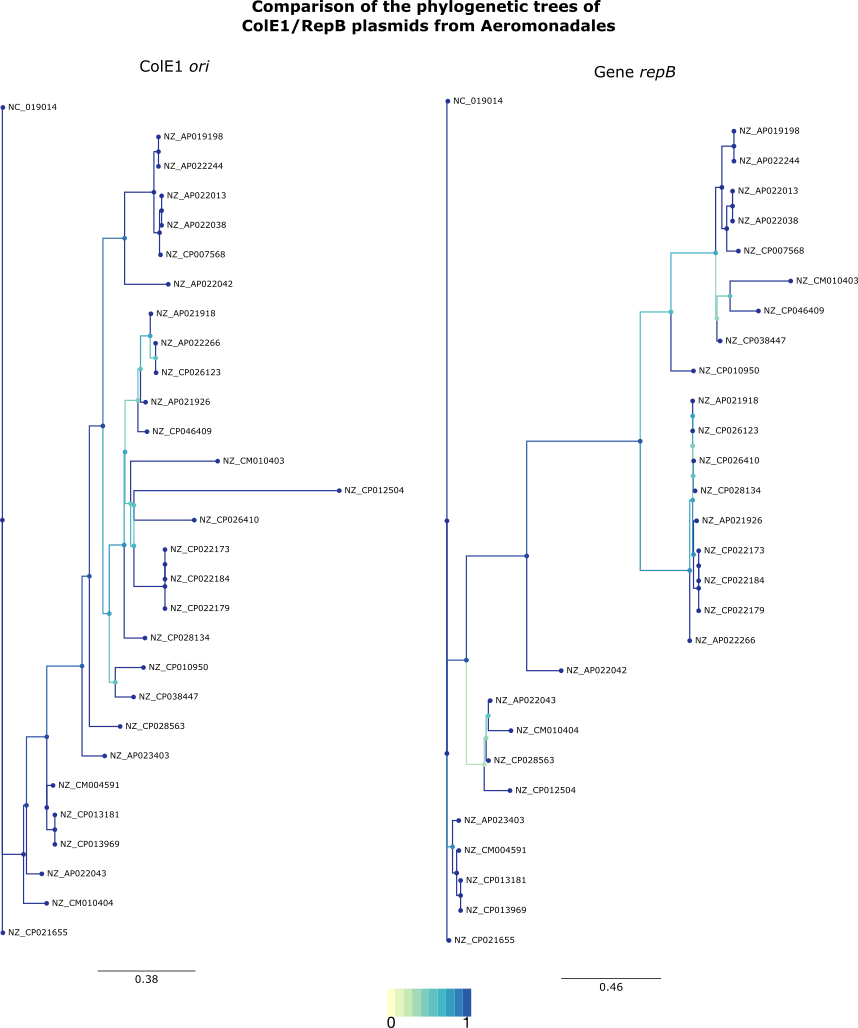

Supplement: S7 Fig — Comparison of the phylogenetic trees of the ColE1 origin of replication (left) (best-fit model JTT+G4) and the gene repB (right) (TN+F+I+G4). The color of the branches represents the comparison metric. The legend is shown at the bottom of the figure. A score of 1 denotes the subtree structure of the node is identical to the subtree structure of its best corresponding node. The figure was performed with the phylo.io tool [77]. (TIFF) [file pgen.1009919.s017.tiff]

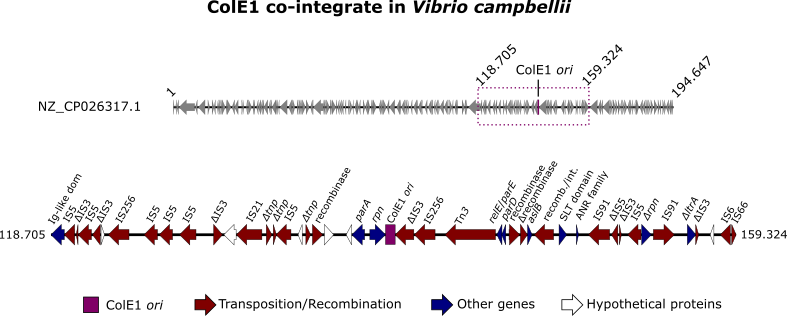

Supplement: S8 Fig — Schematic representation of the ColE1 co-integrate identified in V. campbellii (NZ_CP026317.1). The complete plasmid (194 kb) is represented at the top of the figure. The purple square indicates the genetic environment of the ColE1 origin of replication, which is represented at the bottom of the figure. The ColE1 ori and remaining genes are represented with colored squares and arrows, being the legend at the bottom of the figure. (TIFF) [file pgen.1009919.s018.tiff]

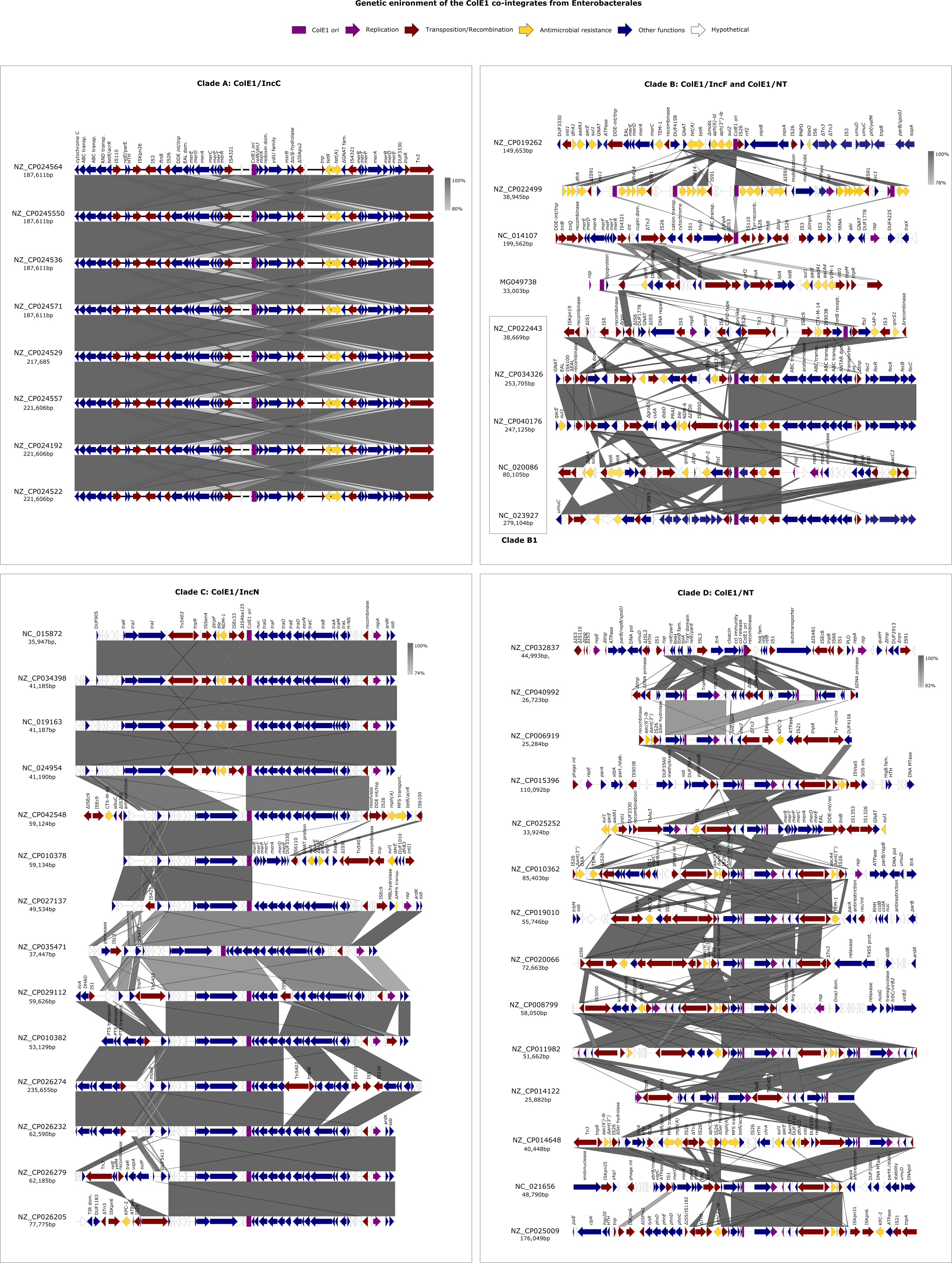

Supplement: S9 Fig — Schematic representation of the genetic environment of the ColE1 ori in the most-represented clades of ColE1 co-integrates from Enterobacterales (Fig 4). The ColE1 ori and remaining genes are represented with colored squares and arrows, being the legend at the top of the figure. The Accession Number and size of each plasmid is indicated at the middle of the figure. (TIFF) [file pgen.1009919.s019.tiff]

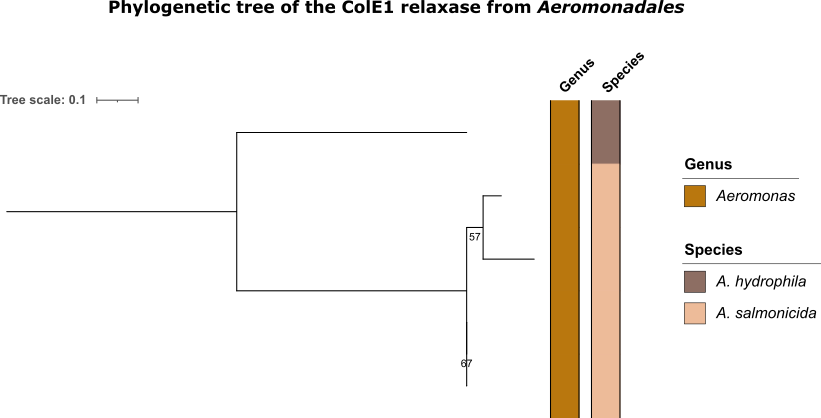

Supplement: S10 Fig — Phylogenetic tree of the ColE1 relaxases from Aeromonadales. The colors of the first column represent the Genus and the second column represents the Species. The legend is at the right of the figure. The bootstrap values are indicated with a number next to each node. The phylogenetic tree was inferred following the best-fit model, JTT+F+G4. (TIFF) [file pgen.1009919.s020.tiff]

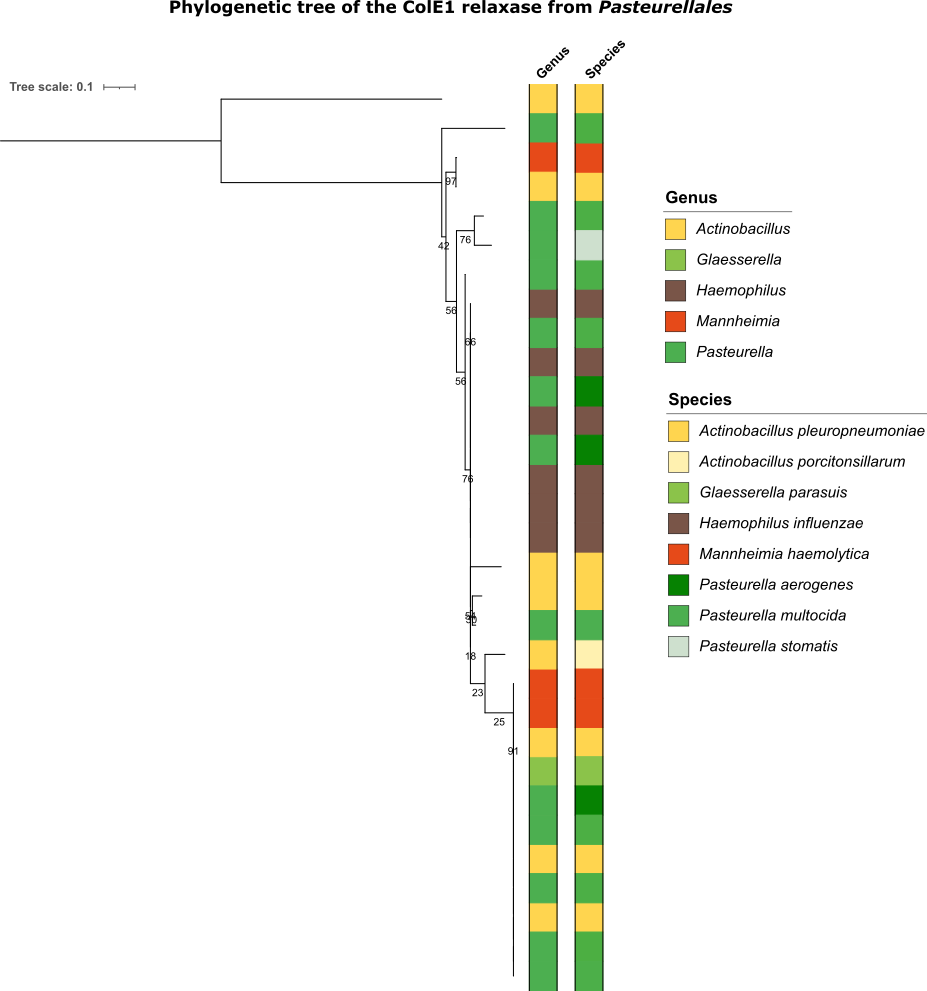

Supplement: S11 Fig — Phylogenetic tree of the ColE1 relaxases from Pasteurellales. The colors of the first column represent the Genus and the second column represents the Species. The legend is at the right of the figure. The bootstrap values are indicated with a number next to each node. The phylogenetic tree was inferred following the best-fit model, VT+G4. (TIFF) [file pgen.1009919.s021.tiff]

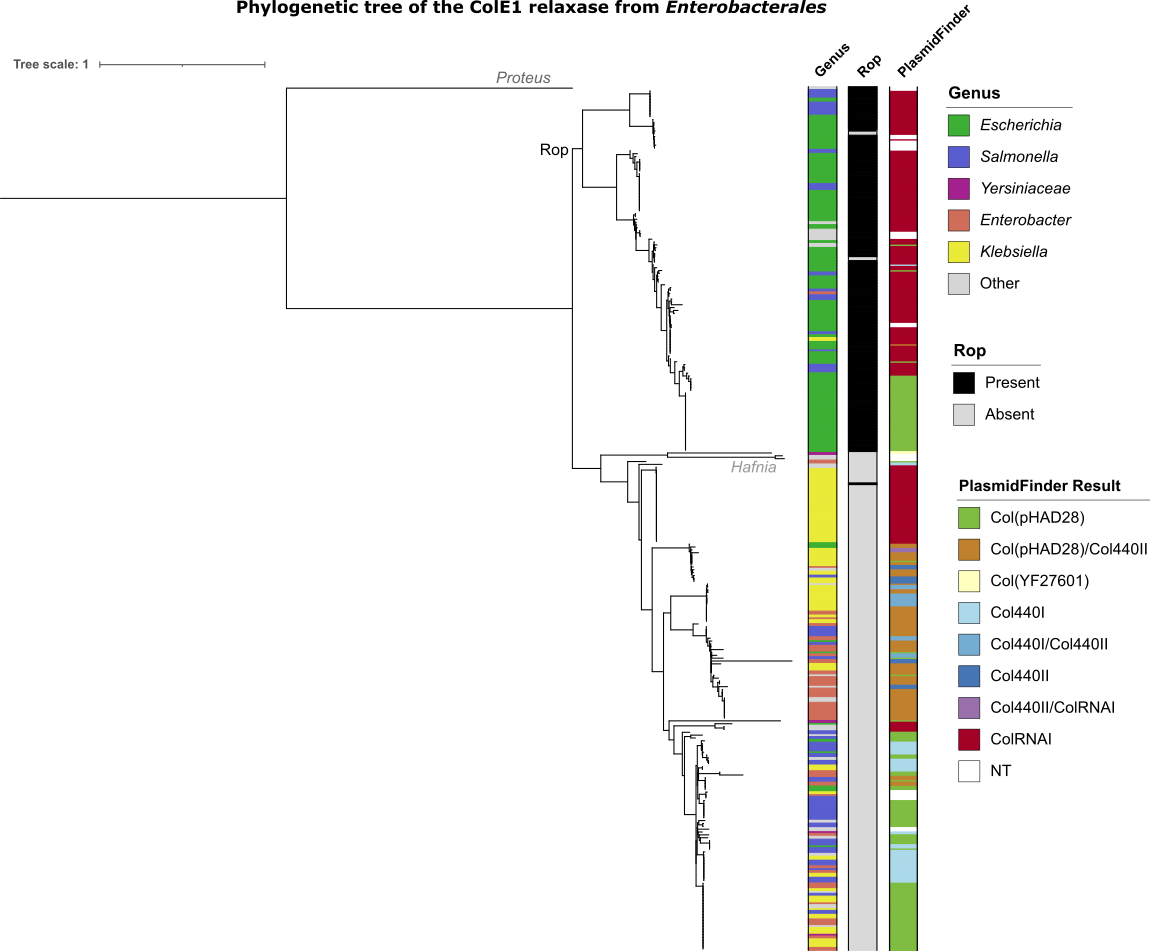

Supplement: S12 Fig — Phylogenetic tree of the ColE1 relaxases from Enterobacterales. The colors of the first column represent the Genus, the second column represents the presence or absence of rop and the third one indicates the PlasmidFinder result. The legend is at the right of the figure. The phylogenetic tree was inferred following the best-fit model, JTT+F+I+G4. (TIFF) [file pgen.1009919.s022.tiff]

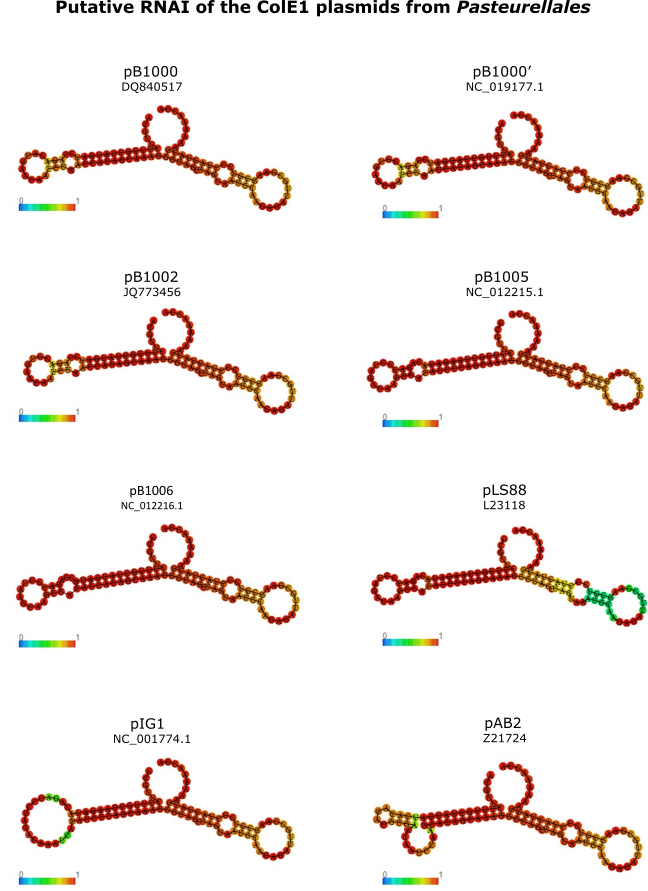

Supplement: S14 Fig — Schematic representation of the secondary structure of the putative RNA I from eight ColE1 plasmids from Pasteurellales. The name and Accession Number of each plasmid is indicated at the top of each sequence. The color of the nucleotides indicates the base-pair probabilities, from 0 to 1, being the legend next to each sequence. The secondary structure and probabilities were inferred with RNAfold WebServer. (TIFF) [file pgen.1009919.s024.tiff]

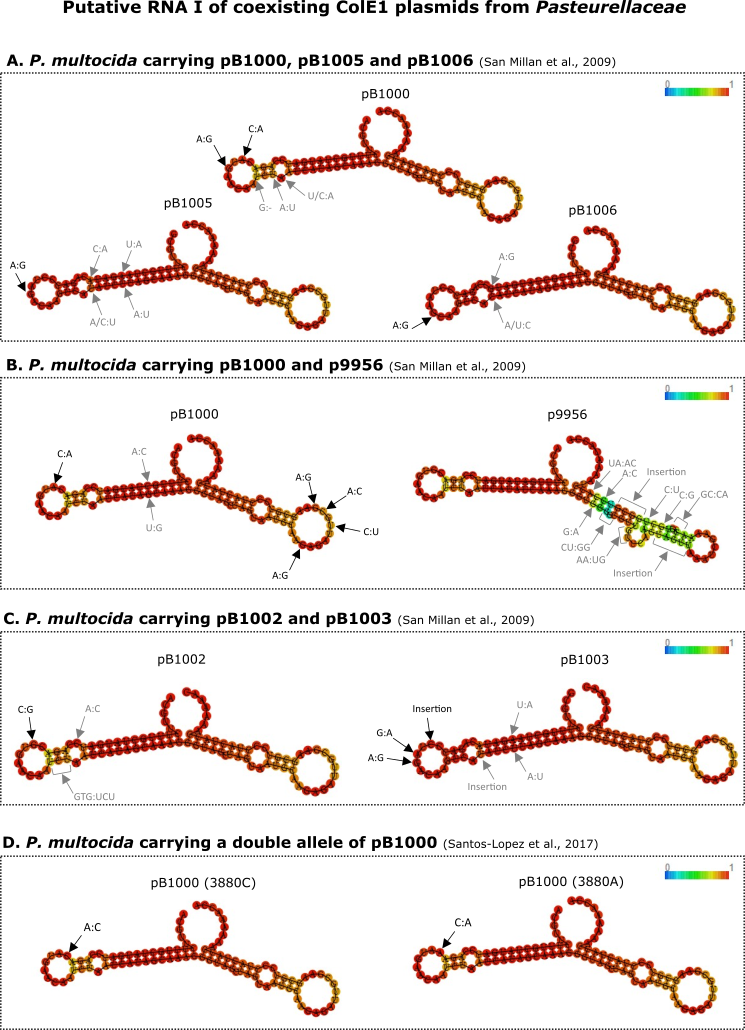

Supplement: S15 Fig — Schematic representation of the secondary structure of the putative RNA I from different ColE1 plasmids from Pasteurellales described coexisting within the cell. The different plasmid combinations are represented separated in the boxes A, B, C and D, being indicated the Species isolate, the plasmid names and the published reference. The Accession Number of each plasmid is indicated at the top of the sequence. The letters and arrows show the dissimilarities identified among the coexisting plasmids, in black those affecting the loop and in grey those affecting the stem. The color of the nucleotides indicates the base-pair probabilities, from 0 to 1, being the legend at the top right of each box. The secondary structure and probabilities were inferred with RNAfold WebServer. (TIFF) [file pgen.1009919.s025.tiff]
